# Explorative Analysis of Antioxidant, Anti-Inflammatory, and Intestinal Barrier Protective Effects of In Vitro Digested Chickpea- and Dark Chocolate-Based Snack: Insights from Caco-2 and THP-1 Cell Models

**DOI:** 10.3390/antiox14070823

**Published:** 2025-07-04

**Authors:** Gaia de Simone, Laura Bonfili, Anna Maria Eleuteri, Laura Bordoni, Rosita Gabbianelli

**Affiliations:** 1 School of Advanced Studies, University of Camerino, 62032 Camerino, Italy; gaia.desimone@unicam.it; 2 Unit of Molecular Biology and Nutrigenomics, School of Pharmacy and Health Products, University of Camerino, 62032 Camerino, Italy; 3 School of Biosciences and Veterinary Medicine, University of Camerino, Via Gentile III da Varano, 62032 Camerino, Italy; laura.bonfili@unicam.it (L.B.); annamaria.eleuteri@unicam.it (A.M.E.)

**Keywords:** healthy snacks, nutrigenomics, antioxidant activity, inflammation, barrier integrity, functional food

## Abstract

Chickpeas are used as alternative protein sources in healthy snacks due to their bioactive compounds beneficial for gut health. Combining chickpeas with dark chocolate improves palatability and may enhance biological functionality, although mechanistic evidence is still limited. In this explorative research, we evaluate the nutrigenomic, antioxidant and anti-inflammatory properties of a chickpea and chocolate snack using in vitro Caco-2 (colon adenocarcinoma cells) and THP-1 (monocyte-derived macrophages) models. The total polyphenol content and antioxidant activity were measured after in vitro digestion (30.30 mg/mL to 1.9 mg/mL). Caco-2 epithelia and THP-1 were pre-treated for 4 days (2 h/day) with high (15.1 mg/mL) or low (3.8 mg/mL) concentrations of digests. Inflammation was induced for 3 h by LPS (Lipopolysaccharides) and IL-1β (Interleukin-1β). Transepithelial electrical resistance (TEER) was measured to assess barrier integrity. Gene expression related to tight junctions and inflammation was analysed using qPCR (quantitative polymerase chain reaction). Chocolate and snack digests showed the highest total polyphenol content and 2,2-diphenyl-1-picrylhydrazyl activity. Barrier integrity improved with all treatments. Chickpea upregulated tight junction gene expression. Chickpea and chocolate reduced IL-1β expression in both cell types. In THP-1, the chocolate and the snack upregulated CD206 (mannose receptor C-type 1) expression. IL-10 increased with all treatments. These results pave the way for future research that may support the potential use of this snack as a functional food with antioxidant, gut-protective and anti-inflammatory effects.

## 1. Introduction

In modern nutrition, snacks have gained widespread popularity as a food choice, especially in the context of a fast-paced lifestyle. Although the rise in snack consumption is more gradual in developing countries than in Western nations, the market continues to expand steadily [1]; in fact, as Michelle Lieszkovszky said in a 2020 interview, “snacking is becoming a lifestyle” [2]. Simultaneously, the current food industry is challenged to meet the demands of health-conscious consumers seeking foods with functional health benefits, and to address sustainability and innovation challenges by—for instance—developing functional foods.

Within this context, the development of “healthy snacks” has emerged as a rapidly expanding global trend, offering a convenient and efficient way to deliver nutrients and bioactive compounds with proven health-promoting properties [1].

With the growing demand for sustainable alternative protein sources and the need to address the low consumption of legumes, pulses are increasingly being incorporated into functional food products—particularly “healthy snacks”—either as a primary ingredient or as a functional component designed to provide additional health benefits [2].

Regular consumption of legumes has been associated with reduced low-grade inflammation and improved immune function, supporting overall health [3,4,5]. Nutritionally, legumes are low in total fats and calories, yet rich in monounsaturated and polyunsaturated fatty acids (MUFAs and PUFAs), fibre, plant sterols, vitamins, minerals, and have a low glycaemic index (GI) [6,7].

Their high polyphenol content contributes to their antioxidant potential, offering protection against conditions such as cardiovascular disease, osteoporosis, gastrointestinal disorders, and neurodegeneration [8,9,10].

Generally, polyphenols are believed to exert these effects primarily through their antioxidant activity, acting as radical scavengers, modulating immune responses and cellular inflammation [11]. For these reasons, plant-based diets are increasingly incentivised as health-promoting and environmentally sustainable options [12,13]. Among legumes, chickpeas (*Cicer arietinum* L.) stand out due to their high nutritional value [14]. Their inclusion in food products is rising, supported by evidence of antioxidant, anti-inflammatory, and antifungal properties [15,16,17,18]. In fact, recent findings suggest that chickpeas may support gut health by reducing inflammation, supporting the microbiota, and enhancing epithelial barrier function [10,19,20,21].

Despite these recognised benefits, it is well known that legume consumption remains relatively low [22]. Consequently, there is a persistent need to develop strategies that encourage greater legume consumption, which could lead to better health outcomes and a more sustainable food supply [23].

The incorporation of chickpeas into snack formulations has attracted increasing interest, especially in response to the growing demand for convenient and health-oriented foods. However, their sensory acceptance remains limited due to their characteristic taste. To address this, recent approaches have focused on combining legumes with other functional ingredients to improve both nutritional value and palatability. In this context, dark chocolate represents a promising complementary ingredient, not only for its potential to enhance flavour but also for its well-documented health benefits [24]. Notably, in recent years, there has been a marked increase in both the production of and research on functional chocolates, highlighting their relevance in the development of novel health-promoting snack products [24].

Chocolate, derived from the processing of cocoa (*Theobroma cacao* L.), is a globally beloved product enjoyed by people of all ages due to its appealing sensory qualities.

Cocoa is known to be rich in polyphenols, which give it strong antioxidant properties [25]. Many in vitro studies have focused on these antioxidant effects and the beneficial properties of cocoa [26,27]. Research has shown that the regular consumption of cocoa (up to 20 g every three days) can help to reduce inflammation and may be beneficial for heart health, cancer prevention. and the immune system. Specifically, it can affect the body’s inflammatory responses and improve immune function in the gut, showing promise as a food with immune-regulating abilities [9,25].

Given these findings, the development of functional chocolates—such as chocolates enriched with bioactive compounds from other functional foods (e.g., pulses)—is gaining significant attention in the scientific community. This approach not only enhances the nutritional value of chocolate but also improves the taste of other functional foods [24].

The primary aim of this study was to explore the nutrigenomic and anti-inflammatory properties of bioactive molecules found in chickpea- and dark chocolate-based snacks after they undergo in vitro gastrointestinal digestion. To successfully achieve this goal, we evaluated the potential usefulness of Caco-2 cells (colon adenocarcinoma cells) as a model for the intestinal epithelium, using trans-well systems to simulate the small intestinal epithelium for transport studies. The barrier properties of Caco-2 were assessed by measuring transepithelial electrical resistance (TEER) and the transport of water-insoluble probes, such as Lucifer Yellow (LY), to assess the epithelial integrity. Despite several protocols describing the Caco-2 trans-well model [21,28,29,30,31], the reproducibility of efficient intestinal epithelium appears to be challenging [29,32,33,34,35,36]. Thus, we tested several conditions to provide additional information on how to reproduce this model.

Furthermore, we used THP-1 cells, an established model for studying monocyte and macrophage functions [5], to assess whether the in vitro digested chickpea- and dark chocolate-based snacks could stimulate the THP-1 immune response by inducing macrophage polarisation towards either a pro-inflammatory or anti-inflammatory phenotype.

## 2. Materials and Methods

### 2.1. Sample Preparation

Fertitecnica Colfiorito, a company based in Colfiorito, Italy, kindly provided us with chickpeas, 60% dark chocolate, and Cioccorito (a snack made from equal parts chickpeas and dark chocolate, 50%/50% *w*/*w*). The process of producing roasted chickpeas involved several carefully controlled steps. First, the raw material went through a magnetic check to ensure high quality. After that, raw materials were soaked in water to hydrate. Once they were ready, they were cooked for 70–90 min at a low temperature of 15–20 °C. The cooking process was followed by a drying phase, using hot air at around 120–130 °C for about 15–20 min. Once dried, the chickpeas were sifted and cooled with cold air. Finally, they were coated in chocolate using a basting technique. The final touch was polishing them with the carnauba wax to give them a nice, shiny finish.

### 2.2. In Vitro Digestion

The in vitro digestion process followed a method developed by Brodkorb and his team [37]. To begin, 1 g each of puffed chickpea, dark chocolate, and the snack was mixed individually with a simulated oral fluid solution for 2 min at 37 °C. This oral fluid contained the enzyme salivary amylase (S-A1031-5KU, Sigma–Aldrich, St. Louis, MO, USA) at a final activity of 75 U/mL to mimic the human oral fluid.

After 2 min, the second step was the stomach phase. Then, 2 mL of a simulated gastric fluid (SGF)—which included an electrolyte solution, pepsin, and gastric lipase (S-L3126-25G, Sigma–Aldrich, St. Louis, MO, USA)—was mixed with the oral fluid of the previous step at a ratio of 1:1 (*v*/*v*). All the enzymes in the SGF achieved a final activity of 2000 U/mL and 60 U/ml.

The acidity was also adjusted by adding HCl to bring the pH to 3, emulating the environment within the human stomach. This mixture was then incubated for 2 h at 37 °C, gently shaken with a Julabo SW-20C shaking water bath to simulate the digestive process.

Once the stomach phase was performed, the gastric chyme was then diluted 1:1 (*v*/*v*) with the simulated intestinal fluid (SIF) solution. According to this, 4 mL of the stomach was mixed with 4 mL of SIF, which contained an electrolyte mix and an enzyme called pancreatin (with a final trypsin activity of 100 U/mL) (S-P1750-25G, Sigma–Aldrich, St. Louis, MO, USA), mimicking intestinal digestion. Then, 1M NaOH was used to adjust the pH to 7. The samples were incubated for another 2 h at 37 °C.

After digestion was completed, the samples were centrifuged at 4000× *g* for at least 30 min at room temperature, and the supernatant was filtered using Millex-GP 0.22 μm polyethersulphone syringe filters (S-SLGS033SS, Sigma–Aldrich, St. Louis, MO, USA) and used for the experiments. The solid part was stored at −80 °C before its application in other analyses [37].

### 2.3. Total Polyphenol Content (TPC)

Although the determination of polyphenols is hampered by their structural complexity, there are many methods for determining the total amount of polyphenolic compounds (TPC) in plants [38]. Colourimetric or biochemical methods for the total quantification of polyphenols are very useful and reliable when quantitative information on the entire set of phenolic compounds is required. The conceptual basis of the method was to quantify the total concentration of phenolic hydroxyl groups in the extract being analysed, irrespective of the individual molecules in which they are found [39]. In this study, the TPC was measured by means of a colourimetric assay using the reaction that leads to the reduction in the Folin–Ciocalteu Reagent to a blue pigment in an alkaline environment, as previously described [40]. Briefly, the digested samples were tested at different concentrations presented as the amount of original digested food product (mg) solubilised in the digestion fluids (mL): 30.3 mg/mL, 15.1 mg/mL, 7.6 mg/mL, 3.8 mg/mL, and 1.9 mg/mL. An aliquot of the previously diluted samples was mixed with Folin reagent in a 96-well microplate and maintained in agitation for 5 min at room temperature. Then Na_2_CO_3_ and distilled water were added. Subsequently, the samples were incubated for 120 min at room temperature in the dark. Then, absorbance was read using a spectrophotometer (FLUOstar Omega, BMG LABTECH’s, Ortenberg, software version 1.30) at 760 nm against a blank. The TPC was then expressed as mg gallic acid equivalents (GAE) per mL of digested sample. All digested samples were analysed in quadruplicate.

### 2.4. DPPH Antioxidant Capacity Assay

The DPPH test was used to estimate the antioxidant activity of the digests based on spectrophotometric measurements of the capacity of antioxidants to scavenge 2,2-diphenyl-1-picrylhydrazyl (DPPH) radicals [41], according to this reaction: DPPH. +AH → DPPH-H +A. A reduction in absorbance corresponds to a decrease in DPPH concentration, which occurs due to the transfer of electrons from the antioxidant compounds [42]. The diluted digested samples were tested at different concentrations presented as the amount of original digested food product (mg) solubilised in digestion fluids (mL): 30.3 mg/mL, 15.1 mg/mL, 7.6 mg/mL, 3.8 mg/mL, and 1.9 mg/mL. The digested samples were then mixed with the 3 mM DPPH working solution in a 96-well microplate and reacted for 20 min in the dark, after which the absorbance was determined at 515 nm with a FLUOstar Omega multi-mode microplate reader (BMG LABTECH’s, Ortenberg, Germany) against a blank. Antioxidant activity was calculated as the percentage of the inhibition of the DPPH radical compared to the control (DPPH without antioxidant). Different concentrations of Trolox, a known antioxidant, were used to prepare a standard curve for DPPH antioxidant activity determination. The Trolox calibration curve was used to express the results as Trolox equivalent (µM TE)/mL of the digested product. All digested samples were analysed in quadruplicate.

### 2.5. ABTS Antioxidant Capacity Assay

In this assay, the stable-coloured cation radical (Ry+) is generated by the oxidation of the diammonium salt of 2,2′-azino-bis (3-ethylbenzothiazoline-6- sulphonic acid) (ABTS) by means of a solution of potassium persulphate (K_2_S2O_8_). This cation radical (ABTSy+) has an absorption peak at 734 nm. Antioxidant compounds (AOH), which can transfer a hydrogen atom or electron to the cation radical, cause discolouration of the solution. The decrease in the peak at 734 nm of the cation radical (ABTSy+) after a predetermined incubation time (10–15 min maximum) was then analysed by UV-Vis spectrophotometry. The absorbance decrease (decolourisation) was proportional to the antioxidant charge in the sample. Briefly, for the ABTS assay, a fresh stock solution was prepared by mixing 7.4 mM ABTS and 2.6 mM potassium persulfate in equal amounts, which were kept for 12 h in the dark. Using the stock solution, a fresh ABTS working solution was prepared by diluting 1 mL of the stock solution with 40 mL of distilled water to obtain a 1.1 ± 0.02 units absorbance at 734 nm using a FLUOstar Omega multi-mode microplate reader. The diluted digested samples were tested at different concentrations presented as the amount of original digested food product (mg) solubilised in digestion fluids (mL): 30.3 mg/mL, 15.1 mg/mL, 7.6 mg/mL, 3.8 mg/mL, and 1.9 mg/mL. Then digested samples were mixed with the 7.4 mM ABTS working solution in a 96-well microplate and incubated in the dark for 20 min, followed by absorbance determination at 734 nm against a blank. Different concentrations of Trolox were used to prepare a standard curve for ABTS antioxidant activity determination. The calibration curve was used to express the antioxidant capacity as Trolox equivalent (µM TE)/mL of the digested product. All digested samples were analysed in quadruplicate.

### 2.6. Oxygen Radical Absorbance Capacity (ORAC)

The oxygen radical scavenging capacity (ORAC) test has been widely used to measure the antioxidant activity of nutraceuticals, pharmaceuticals, and foods. The ORAC test measures the classical ability of an antioxidant to quench free radicals via hydrogen donation.

Firstly, a 75 mM phosphate buffer solution, pH 7, was prepared. Subsequently, a fluorescein stock solution was prepared at a concentration of 4.2 mM in phosphate buffer. Starting with the 4.2 mM solution, a stock was prepared at a concentration of 0.08 µM in buffer. The diluted digested samples were tested at different concentrations presented as the amount of original digested food product (mg) solubilised in digestion fluids (mL): 30.3 mg/mL, 15.1 mg/mL, 7.6 mg/mL, 3.8 mg/mL, and 1.9 mg/mL. Then, the digested samples were mixed with 0.08 µM fluoresceine and 150 mM APPH (2,2,-azobis(2-methylpropionamidine) dihydrochloride). The plate was shaken for 10 s and read every 1.5 min for 90 min using a FLUOstar Omega microplate reader with excitation at 485 nm and emission at 530 nm. Different concentrations of Trolox (6.25–50 µM) were used to prepare a standard curve. Final ORAC values were calculated using the regression equation between Trolox and the net AUC and were expressed as µM TE/mL of digested product. All digested samples were analysed in quadruplicate.

### 2.7. Caco-2

The human epithelial cell line Caco-2 is extensively utilised as a model for the intestinal epithelial barrier [43]. Originally derived from a colon carcinoma, the Caco-2 cell line’s most notable advantage is its ability to spontaneously differentiate into a monolayer of cells that exhibit many characteristics typical of absorptive enterocytes, including a brush border layer like that of the small intestine. Caco-2 cells were kindly provided by Prof. Massimo Nabissi (University of Camerino). Caco-2 cells were cultured in Dulbecco’s modified Eagle’s medium (DMEM, ECB7501L, EuroClone S.p.A, Pero, Milan, Italy) supplemented with 10% heat-inactivated Foetal Bovine Serum (FBS, YOURSIAL-FBS-SA, Sigma–Aldrich, St. Louis, MO, USA), 1% L-glutamine (Stable L-Glutamine, 100X, 200 mM, ECB3004D, EuroClone S.p.A, Pero, Milan, Italy), 1% Non-Essential Amino Acids (NEAAs, ECB3054D, EuroClone S.p.A, Pero, Milan, Italy), and 1% penicillin/streptomycin (P/S 30-002-CI, Sigma–Aldrich, St. Louis, MO, USA). Cells were maintained at 37 °C in a humidified atmosphere with 5% CO_2_. The medium was refreshed every 2 days, and cells were passaged when reaching 80% confluence. The passage number used for this study was 18.

### 2.8. Cell Viability Assay

The cytotoxic impact of chickpea, chocolate, and snack digested products was assessed through the 3-(4,5-Di-2-yl)-2,5-ditetrazolium bromide (MTT) assay (Thiazolyl blue tetrazolium bromide 98%, code 158990050, Acros Organic, NJ, USA). In brief, Caco-2 cells were plated in 96-well plates at a density of 1 × 10^4^ cells/well in complete medium and exposed to various dilutions of digested product presented as the amount of the original digested food product (mg) solubilised in digestion fluids (mL) (30.3 mg/mL, 22.7 mg/mL, 15.1 mg/mL, 7.5 mg/mL, 3.8 mg/mL, and 1.5 mg/mL) for 2 h. Following the incubation period, the cells were treated with a 5 mg/mL MTT solution. After 4 h, the absorbance was measured at 550 nm using a FLUOstar Omega multidetector microplate reader (BMG LABTECH’s, Ortenberg). The results were expressed as a percentage (%) of control (cells grown in culture medium only) and were determined from dose–response curves. The experiment was performed in biological quadruplicates.

### 2.9. Intestinal Epithelium Model

To investigate the anti-inflammatory effects of the digested samples, Caco-2 cells were cultured in a trans-well-based system to evaluate how the digested products influence intestinal permeability and reduce inflammation. Firstly, to establish the optimal conditions for an intestinal epithelium model, different densities of seeding were used following the protocol outlined by Fengguang Pana et al. [30] and by Kyeong Jin Kim et al. [21] all with a few modifications.

Caco-2 cells were seeded on non-coated trans-well inserts (0.4 µm pore size, ThinCert^®^, Greiner Bio-one, Frickenhausen, Germany) at a density of 1 × 10^5^ cells/cm^2^ (apical compartment), and 1 × 10^5^ cells/insert (4.25 cm^2^ apical compartment) in a 6-well plate. The cell culture medium was added in both apical (AP) and basal (BL) compartments to facilitate the development of a differentiated intestinal epithelium. The medium was replaced every 2 days to prevent nutrient depletion. The 6-well plates were incubated in an atmosphere of 5% CO_2_ at 37 °C. Cells were cultured for 15 days and for 21 days in trans-wells until differentiation and complete epithelium formation. Pellets of all the densities were collected at time 15 days post-seeding and at time 21 days post-seeding to evaluate the development of the epithelium. Pellets were stored at −80 °C to prevent alteration of the genetic material to be analysed.

### 2.10. Caco-2 Treatments

Chickpea, chocolate, and snack samples were subjected to in vitro gastrointestinal digestion as described above, freshly prepared, and sterilised using a 0.22 μm filter before being added to the culture media (final concentration of 15.1 mg/mL and 3.8 mg/mL) for 2 h/day over 4 consecutive days prior to inducing inflammation following the method outlined by Frontela-Saseta and co-authors [44] with some modifications. The pre-treatments started on the 14th day post-seeding until the 17th day post-seeding. Each day, following the 2 h incubation period with the digested products, the medium was replaced with fresh medium. The pre-treatments were conducted with a 2 h incubation period to mimic the human gastrointestinal residence time. After 4 consecutive days of pre-treatment with the digested products, a short-term inflammatory response was induced by exposing Caco-2 cells to both interleukin 1β (IL-1β) (SRP3083, Sigma–Aldrich, St. Louis, MO, USA) and lipopolysaccharide (LPS) (Sigma–Aldrich, St. Louis, MO, USA) following the method outlined by Lopes do Carmo and co-authors [45]. IL-1β was added only on the basolateral side, and LPS was applied on both sides (apical and basolateral) [45]. In the apical compartment, 2 mL of DMEM with 1% FBS (*v*/*v*) and LPS (10 µg/mL) was added, while in the basolateral compartment, 4 mL of DMEM with 1% FBS (*v*/*v*) and with both LPS (10 µg/mL) and IL-1β (10 ng/mL) was added. The monolayers were incubated for 3 h. A positive control, in which the Caco-2 monolayer was exposed to the inflammatory stimuli for 3 h, followed by the addition of DMEM (no treatment), was also performed. Monolayers only treated with DMEM were used as a negative control (no inflammation and no treatments). The experiment was performed in biological duplicates. The cell pellets were promptly frozen in liquid nitrogen and stored at −80 °C for subsequent analysis. The cell medium was centrifuged at 13,248× *g* for 5 min, and the clear supernatant was aliquoted into new nuclease-free conical tubes and then stored at −80 °C for future use.

### 2.11. Permeability Assay

The permeability of the Caco-2 monolayer was determined by measuring the transepithelial electrical resistance (TEER), reflecting the integrity of the epithelium, because TEER quantifies the electrical resistance across the epithelial cell layer, which is directly related to the tightness of the tight junctions between cells. To assess the evolution of the intestinal epithelium formation, the barrier integrity was measured using Millicell^®^ ERS (Electrical Resistance System) Voltohmmeter (Millipore, Burlington, MA, USA) in the epithelium with the density of 1 × 10^5^ cells/insert and 1 × 10^5^ cells/cm^2^ after 24 h, 4, 7, 10, 15, and 17 days post-seeding. Three values were measured for each well, which were then averaged and expressed as percentages. According to the literature, Caco-2 cells exhibit TEER values ranging from 150 to 400 Ω.cm^2^ when forming an intact monolayer [46,47].

Additionally, TEER values were collected after pre-treatments with digestive products and a post-inflammation induction to evaluate any protective effect of the samples against inflammation.

The final values are expressed as Ohm × cm^2^ based on the following equation: TEER = (R − Rb) × A, where R is the resistance of the filter insert with cells, Rb is the resistance of the filter alone, and A is the growth area of the filter in cm^2^.

Also, the Lucifer Yellow (LY) (Sigma-Aldrich Life Science, St. Louis, MO, USA) assay was used to measure the permeability of untreated Caco-2 multilayers developed at different densities of seeding (1 × 10^5^/cm^2^ and 1 × 10^5^/well). Briefly, the medium from both the AP and BL compartments was collected after 15 days post-seeding and 21 days post-seeding for each density evaluated and stored at −80 °C for further analysis. Caco-2 cells on the AP side were gently washed twice with Phosphate-Buffered Saline (PBS) with Ca^2+^ and Mg^2+^ (Corning, Glendale, AZ, USA). LY at a concentration of 100 µM (in PBS with Ca^2+^ and Mg^2+^) was introduced into the AP compartment, while PBS (with Ca^2+^ and Mg^2+^) was added to the BL compartment. Subsequently, 150 µL of the solution from both compartments was promptly transferred to a 96-well plate, and fluorescence readings were taken using a fluorometer (FLUOstar Omega, BMG LABTECH’s, Ortenberg) at λEx/λEm = 485/520 nm. These measurements were repeated at 30, 60, and 120 min after the addition of the LY solution in the AP. The cells were maintained at 37 °C in a humidified atmosphere containing 5% CO_2_ between each reading. All analyses were conducted in biological triplicates.

### 2.12. THP-1 Cells

Human monocytic THP-1 is a monocyte isolated from the peripheral blood of an acute monocytic leukaemia patient. This line is commonly employed in studies related to immune system disorders, immunology, and toxicology. THP-1 can be induced to differentiate into a macrophage-like phenotype through treatment with either phorbol-12-myristate-13- acetate (PMA), 1α, 25-dihydroxyvitamin D3 (vD3), or macrophage colony-stimulating factor (M-CSF) [5].

THP-1 cells were purchased from Istituto zooprofilattico sperimentale della Lombardia e dell’Emilia Romagna “Bruno Ubertini”. Cells were cultured in Roswell Park Memorial Institute 1640 medium (RPMI-1640, ECB2000L, EuroClone S.p.A, Pero, Milan, Italy) supplemented with 2-mercaptoethanol to a final concentration of 0.05 mM, 20% heat-inactivated FBS (YOURSIAL-FBS-SA, Sigma–Aldrich, St. Louis, MO, USA), 1% L-glutamine (100X, 200 mM, ECB3004D, EuroClone S.p.A, Pero, Milan, Italy), 1% NEAA (ECB3054D, EuroClone S.p.A, Pero, Milan, Italy), 1% P/S (30-002-CI, Sigma–Aldrich, St. Louis, MO, USA), and 1% of sodium pyruvate (ECM0542D, EuroClone S.p.A, Pero, Milan, Italy). The cells were maintained at 37 °C in a humidified atmosphere with 5% CO_2_.

Culture medium was replaced every 2 to 3 days, and cells were passaged when reaching 80% confluence before any experiment. The passage number used for this study was 13.

### 2.13. THP-1 Treatments

Firstly, to induce polarisation of THP-1 cells into the M0 phenotype macrophages, cells were treated with 100 ng/mL phorbol 12-myristate 13-acetate (PMA) for 24 h in a 6-well plate.

After an incubation period of 24 h to allow for complete adhesion of the cells, the daily treatments started.

THP-1 were exposed to chickpea, chocolate, and snack digested product at concentrations of 15.1 mg/mL; moreover, considering that the snack is made up of 50% chocolate and 50% chickpeas by weight, a concentration of 30.3 mg/mL was also tested to compare the effect of the snack with that observed in the individual components.

Chickpea, chocolate, and snack samples were subjected to in vitro gastrointestinal digestion as described above, freshly prepared, and sterilised using a 0.22 μm filter before being added to the culture media (final concentration of 15.1 mg/mL for chickpea chocolate and snack digested product and 30.3 mg/mL for only snack digested product) for 2 h/day over 4 consecutive days. Each day, following the 2 h incubation period with the digested products, the medium was replaced with fresh medium.

THP-1 monolayers, polarised and only treated with RMPI-1640, were used as a negative control. These cells were untreated, but the medium was replaced with only fresh medium each day.

The experiment was performed in biological duplicates. After 4 consecutive days of treatment with the digested products, cell pellets were promptly frozen in liquid nitrogen and stored at −80 °C for subsequent analysis. The cell medium was centrifuged at 13,248× *g* for 5 min, and the clear supernatant was aliquoted into new nuclease-free conical tubes and then stored at −80 °C.

### 2.14. Gene Expression Analysis

Total RNA was isolated from Caco-2 and THP-1 cells using the Total RNA Purification Plus Kit (Norgen Biotek, Thorold, ON, Canada) following the manufacturer’s protocol and quantified using a NanoDrop spectrophotometer (Thermo Fisher Scientific, Monza, Italy). Then, 1 µg of RNA was retrotranscribed to cDNA (complementary DNA) using the PrimeScript RT-PCR Kit (Takara Bio, Göteborg, Sweden). Gene expression analysis was conducted with quantitative real-time PCR (qPCR) on a Biorad CFX96 system, employing TB Green^®^ Premix Ex Taq™ (Takara Bio, Göteborg, Sweden). The qPCR conditions included an initial denaturation for 30 s at 95 °C; after that, 5 s at 95 °C for denaturation and 30 s at 60 °C for annealing/extension were repeated for 40 cycles. Gene expression levels were normalised to β-actin and to Peptidylprolyl Isomerase A (PPIA) using the 2^−∆∆CT^ method. Each assay was performed in technical duplicates, with an inter-run calibrator sample to standardise the results across different amplification plates. Biological duplicates were analysed for each treatment condition.

The genes of interest analysed from Caco-2 cells included the tight junction genes Zonulin 1 (ZO-1), Occludin (OCLN), and Claudin1 (CLDN1); the genes involved in Caco-2 differentiation ALPI Alkaline Phosphatase Intestinal (ALPI), Cytochrome P450 Family 3 Subfamily A Member 4 (CYP3A4), SLC15A1 Solute Carrier Family 15 Member 1 (SLC15A1), Solute Carrier Family 11 Member 2 (SLC11A2), and Sucrase-Isomaltase (SI); and the pro-inflammatory genes interleukin-6 (IL-6), IL-1β, Nuclear Factor Kappa B (NF-κB), Interleukin 8 (IL-8), Monocyte Chemoattractant Protein 1 (MCP1 or CCL2), Chemokine (C-C motif) Ligand 20 (CCL20), and Tumour Necrosis Factor Alpha (TNF-α). Primer sequences used in the study are provided in Supplementary Materials Table S1.

For THP-1 cells, the analysis focused on genes related to inflammatory and anti-inflammatory pathways, including IL-1β, IL-6, IL-8, MCP1, NF-κB, C-type mannose receptor 1 (CD206), interleukin-10 (IL-10), Transforming Growth Factor Beta (TGFβ), Peroxisome Proliferator-Activated Receptor Gamma (PPAR-γ), and Cluster of Differentiation 163 (CD163). All genes evaluated were based on a study carried out by Matacchione G. et al. [48]. The primer sequences used in the study are provided in Supplementary Materials Table S1.

### 2.15. Statistical Analysis

Statistical analysis was performed using SPSS (IBM SPSS Statistics for Windows, Version 29.01.0, Armonk, NY, USA). The results were expressed as mean ± standard deviation. It was verified that the parameters followed a normal distribution. The existence of significant differences among the different parameters in the samples was evaluated using the non-parametric Kruskal–Wallis test. An ANOVA test with Bonferroni’s correction was used to test differences between means. The Bonferroni correction was applied to correct *p* values in multiple comparisons. Moreover, Spearman’s correlation was used to evaluate the presence of a positive linear correlation between the total polyphenol content and antioxidant value. A *p* value < 0.05 was considered significant throughout the study.

## 3. Results

### 3.1. Evaluation of Total Polyphenol Content TPC

The evaluation of the total polyphenol content in chickpea, chocolate, and snack digested samples was measured after in vitro digestion and was conducted using the Folin–Ciocalteu method.

**Figure 1:**
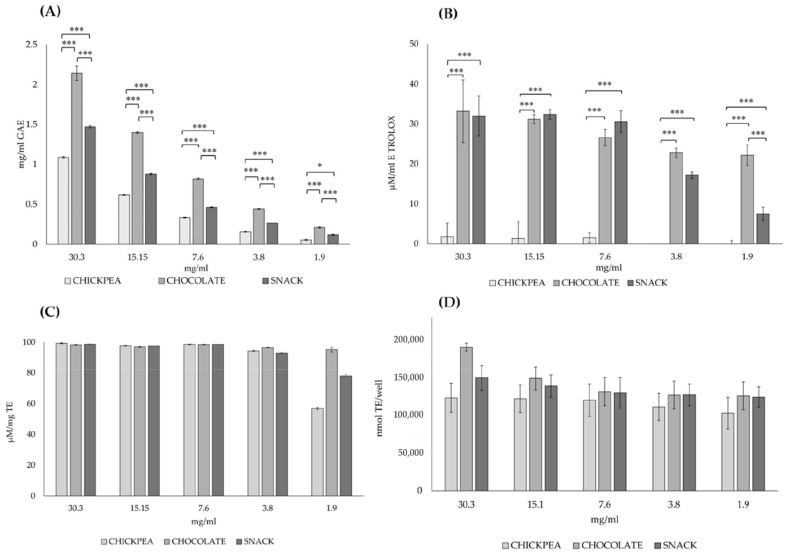
Total polyphenol content and antioxidant capacity of digested product samples. The total polyphenol content (**A**) is expressed as mg of gallic acid equivalents (GAE) per mL of digested product. Antioxidant capacity was assessed using three different assays: DPPH (**B**), ABTS (**C**), and ORAC (**D**). The results from the DPPH and ABTS assays are expressed as Trolox equivalents (TE) in µM per mL, while the ORAC results are expressed as nmol TE per well. All concentrations of the digested product samples tested are obtained as the mean of four replicates. * *p* < 0.05; *** *p* < 0.001 snack vs. chickpea, snack vs. chocolate and chickpea vs. chocolate. An ANOVA test with Bonferroni’s correction was used to test the differences between means.

In the present study, the total polyphenol content in the digested samples, expressed as GAE in mg per mL of digested product (y = 2.8235x + 0.1255, R^2^ = 0.9894), exhibited a clear dose-dependent trend (Figure 1A). Statistically significant differences in total polyphenol content were observed among the digested products when comparing chickpea with chocolate (30 mg/mL *p* = 1.23 × 10^−42^, 15 mg/mL *p* = 7.19 × 10^−37^, 7.6 mg/mL *p* = 6.76 × 10^−28^, 3.8 mg/mL *p* = 2.04 × 10^−18^, 1.9 mg/mL *p* = 1.26 × 10^−9^) and the snack with chocolate across all tested concentrations (30 mg/mL *p* = 5.30 × 10^−34^, 15 mg/mL *p* = 3.81 × 10^−29^, 7.6 mg/mL *p* = 2.87 × 10^−22^, 3.8 mg/mL *p* =3.17 × 10^−11^, 1.9 mg/mL *p* = 0.0004). Furthermore, significant differences were found when comparing snack digested samples with chickpea digested samples at concentrations ranging from 30.3 mg/mL to 3.8 mg/mL (30 mg/mL *p* = 1.47 × 10^−23^, 15 mg/mL *p* = 4.59 × 10^−17^, 7.6 mg/mL *p* = 2.71 × 10^−7^, 3.8 mg/mL *p* = 2.6 × 10^−4^). A lower level of significance was observed when comparing chickpea digested samples with snack digested samples at 1.9 mg/mL (*p* = 0.045).

### 3.2. DPPH Results

The antioxidant activity of all the digested samples, measured by the DPPH assay, was expressed in terms of Trolox equivalent (µM TE)/mL of the digested product (y = 11.159x − 19.348, R^2^ = 1).

The antioxidant activity evaluated with the DPPH assay demonstrated a dose-dependent trend in the digested samples from chocolate and snack matrices, while it was absent in those derived from the chickpea matrix (Figure 1B). Statistically significant differences in antioxidant activity were observed between the digested products when comparing chickpea with chocolate (30 mg/mL *p* = 3.44 × 10^−12^, 15 mg/mL *p* = 8.03 × 10^−14^, 7.6 mg/mL *p* = 9.05 × 10^−11^, 3.8 mg/mL *p* = 1.8 × 10^−12^, 1.9 mg/mL *p* = 6.01 × 10^−12^) and chickpea with the snack (30 mg/mL *p* = 1.33 × 10^−10^, 15 mg/mL *p* = 1.80 × 10^−13^, 7.6 mg/mL *p* = 1.76 × 10^−13^, 3.8 mg/mL *p* = 2.27 × 10^98^, 1.9 mg/mL *p* = 3.6 × 10^−9^).

### 3.3. ABTS Results

Antioxidant activity, measured with ABTS assay, was expressed in terms of Trolox equivalent (µM TE)/mL of digested product (y = 8.1668x − 28.918, R^2^ = 0.9918).

The antioxidant activity showed no dose-dependent trend (Figure 1C). In all concentrations tested, ranging from 30.3 mg/mL to 1.9 mg/mL, no significant variations in antioxidant activity were found between all the digested products.

### 3.4. ORAC Results

Figure 1D shows the antioxidant capacity of all digested products expressed as µM TE/mL of digested product (y = 39411x + 24557, R^2^ = 0.9731). In all the concentrations evaluated, from 30.3 mg/mL to 1.9 mg/mL, no significant differences in antioxidant activity were observed among all the digested products.

### 3.5. Correlation Between Phenolic Content and Antioxidant Activity

A highly significant correlation was found between TPC and ORAC values (provided in Supplementary Materials Table S2) in all digested products. In fact, Spearman’s correlation coefficient (r = 0.572; *p* < 0.001) revealed a moderate positive correlation between TPC and ORAC values, with a strong statistical significance. A similar effect was observed comparing the TPC and antioxidant activity assayed by the ABTS method, with a positive correlation coefficient r = 0.727 (*p* < 0.001). Despite this, no positive and significant correlations were seen comparing TPC and DPPH values (r = 0.262; *p* = 0.63).

### 3.6. Cell Viability

To find the non-cytotoxic dose of the digested products from chickpea, dark chocolate, and chocolate-based snacks for the Caco-2 cells, an MTT assay was performed. The cell viability values referred to each extracted product concentration were compared to the average of the survival naive to calculate the viability percentage for every concentration of extracted product.

**Figure 2:**
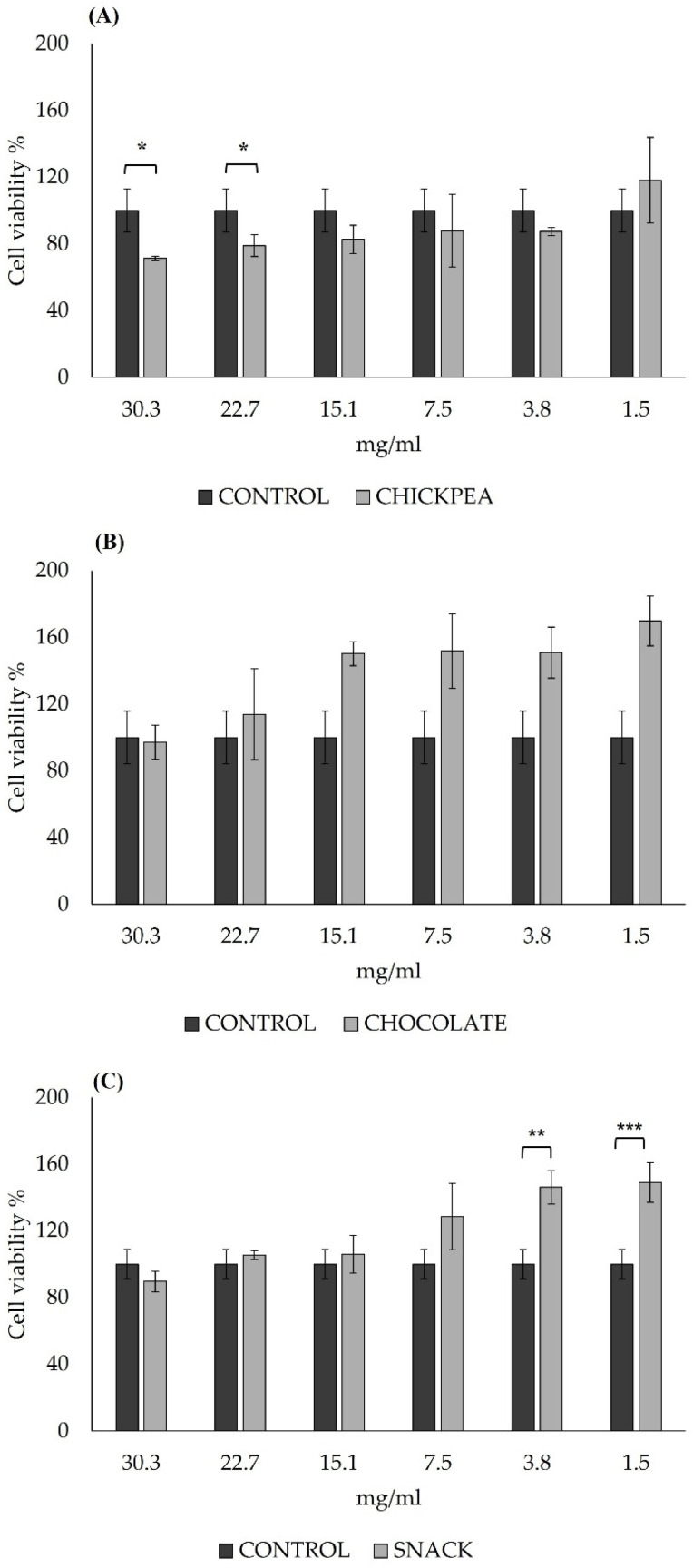
Assessment of cytotoxicity of chickpea (**A**), chocolate (**B**), and snack (**C**) digested products on Caco-2 cells after 2 h exposure to different concentrations (ranging from 30.3 mg/mL to 1.5 mg/mL). Cell viability is expressed as % of control (untreated cells) ± SD for each condition. * *p* < 0.05; ** *p* < 0.01; *** *p* < 0.001 vs. control. ANOVA test with Bonferroni’s correction was used to test differences.

The results from the MTT assay revealed that 2 h exposure to chickpea digested product exhibits no significant decrease in cell viability for the concentrations tested, ranging from 15.1 mg/mL to 1.5 mg/mL (Figure 2A). The MTT assay results of dark chocolate digested product (Figure 2B) showed no cytotoxic effects on Caco-2 cells for the concentrations tested. The snack digested product (Figure 2C) showed no cytotoxic effects on Caco-2 cells for all the concentrations tested. On the contrary, the viability of cells treated with 3.8 mg/mL (*p* = 0.0001) and 1.5 mg/mL (*p* = 0.00003) increased.

Based on the results obtained, there were no cytotoxic concentrations in the digests tested except for the chickpea-derived digest tested at concentrations of 30.3 mg/mL and 22.7 mg/mL. Based on this result, two concentrations, a high (15.5 mg/mL) and a low (3.8 mg/mL) concentration of the products, were chosen to be tested on the intestinal epithelium.

### 3.7. Optimisation of an Intestinal Epithelium Model

With the aim to set the best conditions to explore the anti-inflammatory effect of all digested products on an intestinal epithelium model, our study tests were conducted with two densities: one high (1 × 10^5^/cm^2^) and one low (1 × 10^5^/well), to develop a well-formed intestinal epithelium and to identify which was more suitable for our analysis.

Caco-2 cells were seeded on non-coated trans-well inserts for 15 days and 21 days; the evaluation of the epithelium was performed by analysing the gene expression of the differentiation genes (ALPI, SLC11A2, SLC15A1, and CYP3A4, SI) and of the tight junctions (ZONULIN1, OCCLUDIN, and CLAUDIN1) after 15 days post-seeding and 21 days post-seeding. For genes related to differentiation into mature intestinal enterocytes (ALPI, SLC11A2, and SLC15A1), expression levels initially increased in a time-dependent manner but decreased with extended seeding days. By day 21 after seeding, no significant differences in gene expression levels were observed across all cases evaluated. For ALPI expression and for SLC11A2 expression (the results are shown in Supplementary Materials Figure S1A,C), the results showed an overall *p* value of 0.053, suggesting a potential trend approaching significance. Significantly elevated levels of ALPI were measured in Caco-2 intestinal epithelium after 15 days in both densities (*p* = 0.008 in both cases). In contrast, the expression of SLC11A2 was affected only by the density of 1 × 10^5^/cm^2^ after 15 days of seeding (*p* = 0.031). No significant differences in expression levels were detected for SLC15A1 (the results are provided in Supplementary Materials Figure S1B). No expression of the SI and CYP3A4 genes was detected in any of the samples analysed.

Moreover, to assess the formation of an intestinal epithelium, the gene expression of the tight junctions (ZONULIN1, OCCLUDIN, and CLAUDIN1) was evaluated 15 days after seeding and 21 days after seeding (the results are shown in Supplementary Materials Figure S2A–C). In all cases examined, there was an increased expression of the genes related to tight junction formation after 15 days post-seeding.

Significantly elevated levels of CLAUDIN1 and ZONULIN1 were measured in Caco-2 intestinal epithelium after 15 days in 1 × 10^5^/well (*p* = 0.016 in both cases). In contrast, the expression of OCCLUDIN was affected only by the density of 1 × 10^5^/cm^2^ after 15 days of seeding (*p* = 0.008).

Other parameters that have been most frequently used to assess the permeability of intestinal epithelial cell monolayers and are related to tight junctions are the measurement of either TEER or the apparent permeability (Papp) measured with Lucifer Yellow. No significant differences in permeability were measured between the different densities of seeding with the Lucifer Yellow assay after 15 days of seeding and after 21 days of seeding (the results are shown in Supplementary Materials Figure S3).

In this context, the ohmic resistance, reported in Ω-cm^2^, of the epithelium developed from a density of 1 × 10^5^ cells/cm^2^ and 1 × 10^5^ cells/insert, during a 17-day culture period, was evaluated (the results are shown in Supplementary Materials Figure S4A,B). By studying the expression of genes associated with tight junctions and cell differentiation, it was observed that on day 21, the epithelium started to degrade, with a decrease in the expression of the genes analysed. To obtain further confirmation on the optimal cell density required to develop a well-formed epithelium, TEER values were collected during 17 days of seeding. The results obtained from monitoring TEER over the 17-day seeding period using a density of 1 × 10^5^ cells/cm^2^ showed an irregular pattern of epithelial growth over time. The values indicated solid epithelial formation starting from day 7 (reaching values above 150 Ω·cm^2^), followed by a decline below the threshold considered adequate for a well-formed epithelium. Consequently, epithelium with this density began to degrade after day 10 and was not suitable for further analysis (Figure S4A). On the contrary, the results obtained from monitoring the TEER during the 17-day of seeding showed a regular course of epithelium growth over time, when the epithelium was developed using the density 1 × 10^5^ cells/insert (Figure S4B). In this case, the epithelium started forming as early as day 7, reaching values above 150 Ω-cm^2^. In summary, based on our data regarding gene expression and TEER measurements, we found that, in our experimental settings, most of the monolayers reached differentiation between days 14 and 17.

Based on these results, Caco-2 cells seeded at a density of 1 × 10^5^/well were treated 14 days after seeding to test the anti-inflammatory effect of chickpea, dark chocolate, and snack digested products on the optimised intestinal epithelium model.

### 3.8. Assessment of the Intestinal Barrier Integrity and Expression Levels of Tight Junctions’ Genes After Treatments

Before treating the intestinal epithelium with digests at the two non-cytotoxic concentrations evaluated using the MTT assay (15.1 mg/mL and 3.8 mg/mL), the intestinal epithelium was developed and monitored using a seeding density of 1 × 10^5^ cells/well. A critical prerequisite for starting the pre-treatments was the development of a well-formed epithelium. To verify the formation of a correct intestinal epithelium, the TEER values were checked to ensure that they reached the parameters described in Section 3.7 before starting the experiment (the results are shown in Supplementary Materials Figure S5).

**Figure 3:**
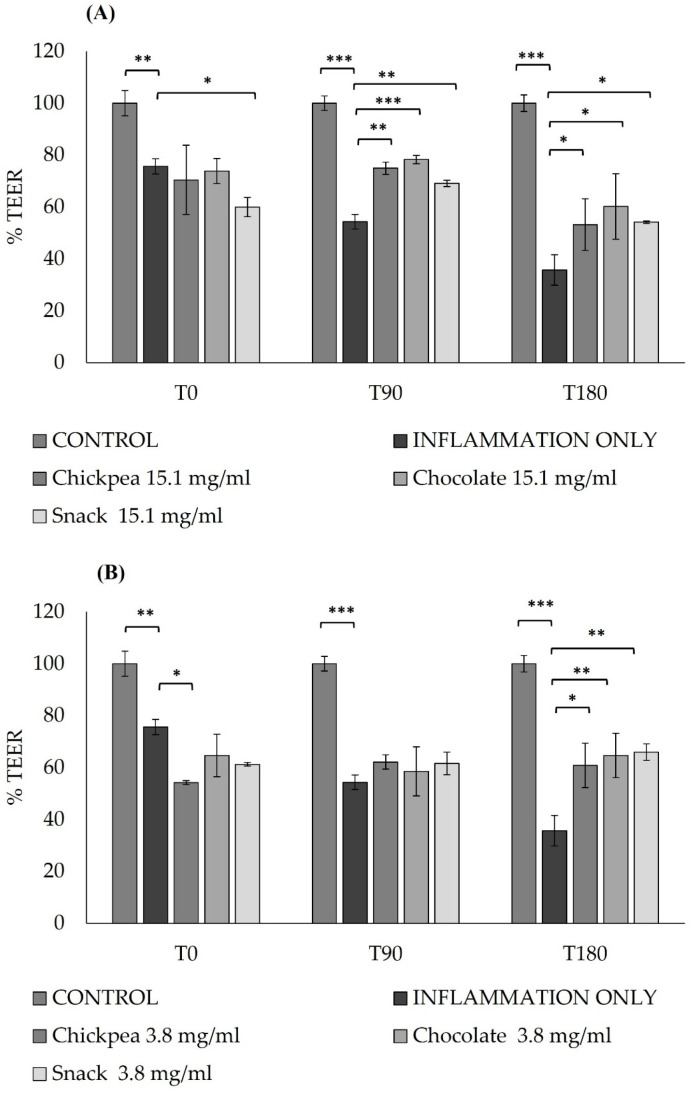
Effect of digested products on intestinal barrier integrity under inflammatory conditions. TEER was measured in Caco-2 monolayers exposed to inflammation (LPS + IL-1β for 3 h, day 17 post-seeding), after treatment with 15.15 mg/mL (**A**) or 3.8 mg/mL (**B**) of digested chickpea, dark chocolate, or snack for 4 days (2 h/day). Results are expressed as % of untreated control (naïve) at T0, T90, and T180. * *p* < 0.05; ** *p* < 0.01; *** *p* < 0.001 vs. inflamed control at corresponding time points. Data are mean ± SD from two independent experiments. ANOVA test with Bonferroni’s correction was used to test differences.

The integrity of the intestinal monolayer after 4 consecutive days of pre-treatment and a short 3 h inflammatory stimulus was applied and monitored with TEER to assess the protective effects of digested chickpea, chocolate, and snack products in both concentrations of 15.1 mg/mL (Figure 3A) and of 3.8 mg/mL (Figure 3B). A significant reduction in the epithelium integrity was measured after 3 h exposure to LPS 10 µg/mL stimulated in the apical department and LPS 10 µg/mL with IL-1β 10 ng/mL in the basolateral department, without treatment, and used as positive controls for a pro-inflammatory condition.

The results obtained showed that the epithelium integrity was significantly protected by the digested products tested at 15.1 mg/mL after 180 min (inflammation only vs. chickpea 15.1 mg/mL *p* = 0.049, inflammation only vs. chocolate 15.1 mg/mL *p* = 0.011, and inflammation only vs. snack 15.1 mg/mL *p* = 0.040) and the digested products tested at 3.8 mg/mL after 180 min (inflammation only vs. chickpea 3.8 mg/mL *p* = 0.010, inflammation only vs. chocolate 3.8 mg/mL *p* = 0.005, and inflammation only vs. snack 3.8 mg/mL *p* = 0.003) and after 3 h inflammatory exposure when compared to the positive control group at the same time points (Figure 3).

**Figure 4:**
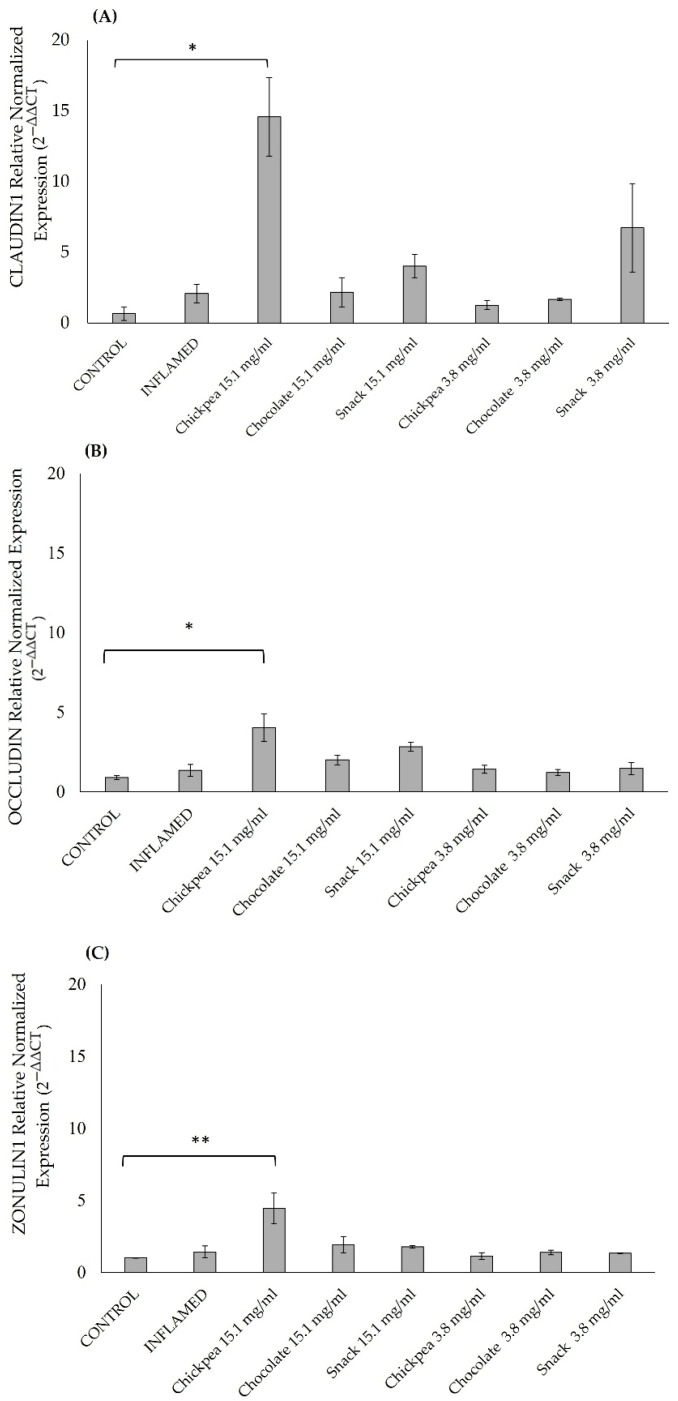
Effects of digested products on expression of *CLDN1* (**A**), *OCLN* (**B**), and *ZO-1* (**C**) genes in Caco-2 cells (17 days post-seeding). Cells were treated with 15.15 or 3.8 mg/mL of digested products for 4 days and then exposed to short-term inflammatory stimuli (LPS 10 µg/mL apical; LPS 10 µg/mL + IL-1β 10 ng/mL basolateral). Gene expression is shown relative to inflamed, untreated control. * *p* < 0.05; ** *p* < 0.01 vs. inflamed control. Values represent mean ± SD from two independent experiments. ANOVA test with Bonferroni’s correction was used to test differences between means.

To further confirm the potential protective effect of the digested samples on intestinal permeability, the expression levels of the tight junctions ZO-1, CLAUDIN1, and OCCLUDIN were also measured in Caco-2 cells after the pre-treatments with the digested samples mentioned above and the subsequent short 3 h inflammatory stimulus. A significant increase in the expression levels was detected after chickpea digested product treatment at 15.1 mg/mL when compared to the control in the Claudin1 gene (Figure 4A) with a *p* = 0.039, the Occludin gene with a *p* = 0.013, (Figure 4B), and the Zonulin1 gene with a *p* = 0.009, (Figure 4C).

### 3.9. Expression Levels of Pro and Anti-Inflammatory Genes in Caco-2 and THP-1 Cells

**Figure 5:**
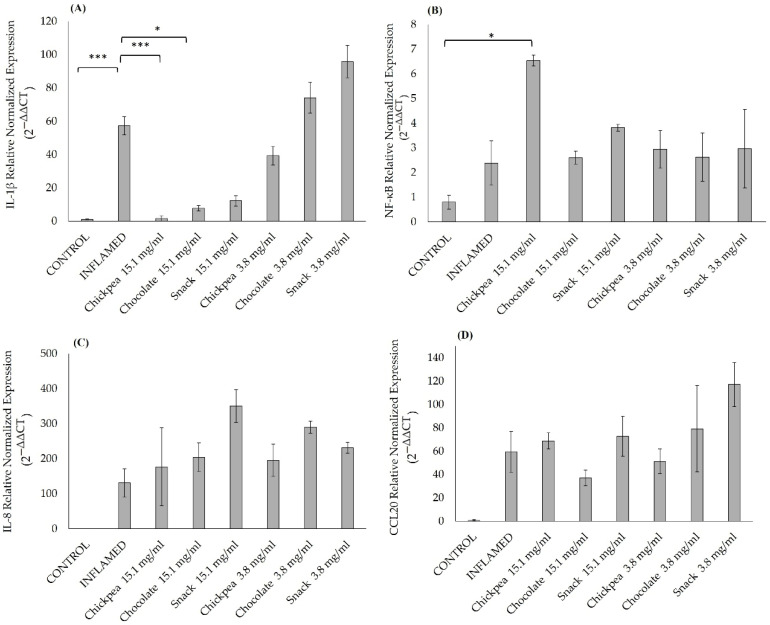
Effects of 3 h inflammatory stimuli on IL-1β (**A**), NF-κB (**B**), IL-8 (**C**), and CCL20 (**D**) gene expression levels in a Caco-2 monolayer epithelium measured by qPCR after chickpea, dark chocolate, and snack digested product treatments at 15.1 mg/mL and 3.8 mg/mL for four consecutive days (from the 14th day post-seeding to the 17th day post-seeding). *** *p* < 0.001; * *p* < 0.05 vs. control. Results of controls, inflamed, and samples treated with digested product are expressed as mean ± SD from two independent experiments. ANOVA test with Bonferroni’s correction was used to test differences between means.

The results showed that digested chickpea, dark chocolate, and snack products influenced the inflammatory response differently depending on the cell lines used. In Caco-2 cells, a statistically significant decrease in IL-1β expression was observed after pre-treatment with the chickpea digested product at 15.1 mg/mL (*p* = 0.0004) and the chocolate digested product at 15.1 mg/mL (*p* = 0.040) (Figure 5A). A significant increase in NF-kB expression was induced by the chickpea digested product at 15.1 mg/mL (*p* = 0.022) (Figure 5B). Conversely, IL-8 (Figure 5C) and CCL20 (Figure 5D) expressions remained unaffected at all the concentrations tested.

**Figure 6:**
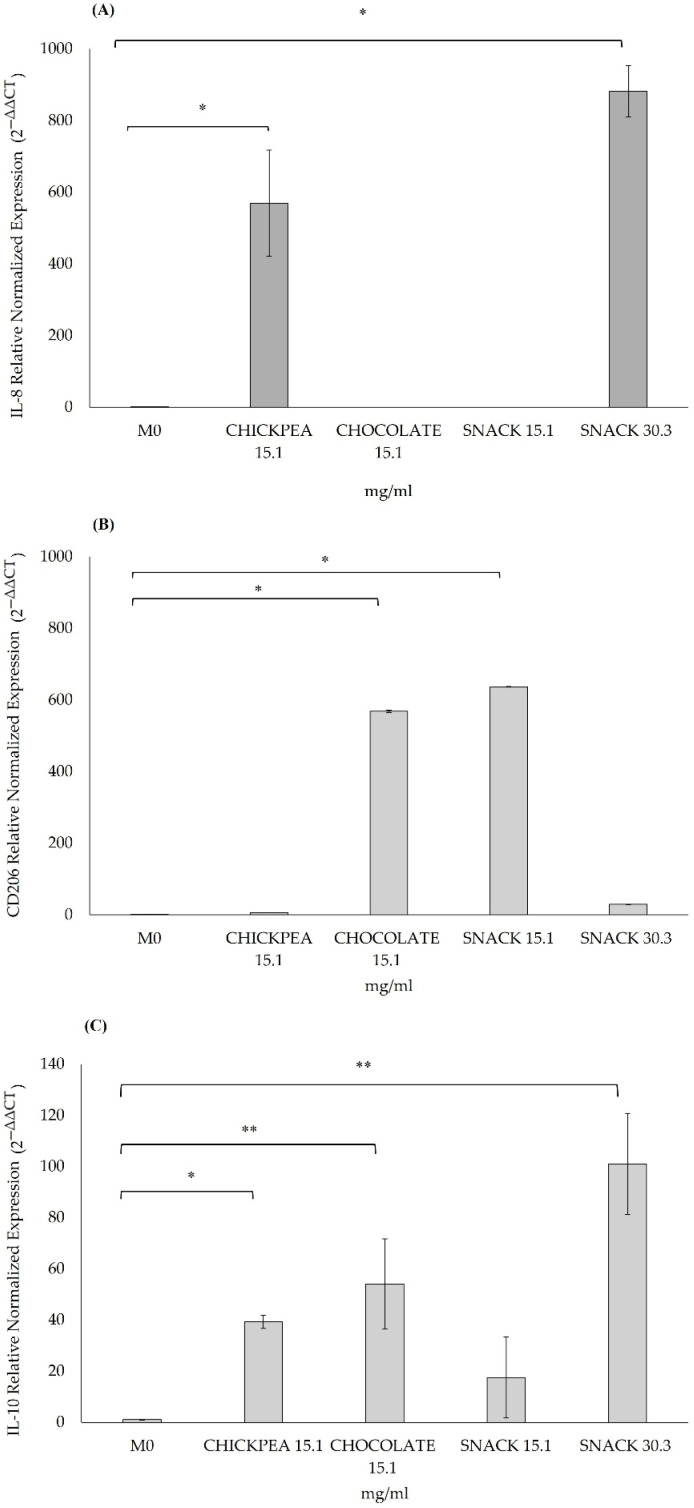
Effects on pro-inflammatory genes, IL-8 (**A**), and anti-inflammatory genes, CD206 (**B**) and IL-10 (**C**). Gene expression levels in a THP-1 monolayer measured by qPCR after chickpea, dark chocolate, and snack digested product treatments at 15.1 mg/mL and 30.3 mg/mL, only snack digested sample, for four consecutive days. ** *p* < 0.01; * *p* < 0.05 vs. control (no treated cells, M0). Results of controls and treated with digested product samples are expressed as mean ± SD from two independent experiments. Existence of significant differences among different parameters in samples was evaluated using non-parametric Kruskal–Wallis test.

In THP-1 immune cells, a statistically significant upregulation of IL-8 (Figure 6A) gene expression was observed in cells exposed to 15.1 mg/mL of the chickpea digested product and 30.3 mg/mL of the snack digested product compared to untreated M0 cells (*p* < 0.05).

Concerning anti-inflammatory markers in THP-1 cells, a statistically significant upregulation in the expression of CD206 genes (Figure 6B) was detected in cells treated with both chocolate and snack digested products at a concentration of 15.1 mg/mL (*p* = 0.008, *p* = 0.003, respectively). Additionally, a significant increase in IL-10 (Figure 6C) gene expression levels was observed in cells treated with chickpea (*p* = 0.017) and dark chocolate digested products (*p* = 0.006) at 15.1 mg/mL, and with snack digested products at 30.3 mg/mL (*p* = 0.002).

## 4. Discussion

Persistent inflammation and oxidative stress play a critical role in the development of chronic inflammatory conditions in the immune system.

Legumes, such as chickpeas, help to reduce this effect thanks to their high levels of phenolic content, which provide antioxidant and anti-inflammatory benefits [49].

In this study, we showed that a puffed chickpea snack enriched with dark chocolate is enriched in polyphenol levels and antioxidant activity. After in vitro digestion, all digested products were non-cytotoxic at the tested concentrations. They maintained intestinal barrier integrity under inflammatory conditions and modulated the expression related to tight junction and inflammation genes in both Caco-2 and THP-1 cells.

Based on the antioxidant activity and capacity, the results showed that the TPC of all digests influenced the ABTS and ORAC values. The antioxidant capacity, as measured by ABTS and ORAC assays, was positively correlated with the TPC. Specifically, we found a correlation of *r* = 0.727 for ABTS and *r* = 0.572 for ORAC, indicating that higher TPC values led to stronger antioxidant responses. No significant correlation was observed between TPC and DPPH values (*r* = 0.262), suggesting assay-specific responses. In particular, a positive correlation was found between the TPC and antioxidant activity measured by the ORAC method (*r* = 0.572) and the ABTS assay (*r* = 0.727). However, no significant correlation was observed between the TPC and the DPPH assay (*r* = 0.262). The results obtained agree with those of the study by are consistent with Rajagukguk et al. [50], who examined the total polyphenol content in a dark chocolate and chickpea snack bar. The TPC was found to significantly influence ORAC, DPPH, and ABTS antioxidant activity values, with reductions in the TPC leading to corresponding decreases in these measurements. It has been reported that the TPC significantly influenced antioxidant capacity in chickpea–dark chocolate snack bars across several assays [50].

Moreover, other studies on chickpea-based products reported similar trends. Xiao Y. et al. [51] observed positive correlations between the TPC and antioxidant activities (ABTS, DPPH, ORAC, and PCL). A similar highly significant correlation between the TPC and antioxidant assays (ABTS and ORAC) in chickpea samples was found by Sanchez-Magana, whose positive linear correlation (*r* = 0.991 and *r* = 0.972, respectively) can be considered extremely significant [52]. This reduction may be attributed to the heat-related degradation or transformation of certain compounds.

Importantly, DPPH and ORAC measure different antioxidant mechanisms—electron transfer versus hydrogen atom transfer, respectively—thus reflecting the activity of distinct phenolic subgroups. Previous studies have also explored each ingredient individually. However, the lack of correlation with the DPPH assay in our results aligns with the findings of Xu et al. [53], who observed a 10–35% reduction in DPPH activity after chickpeas soaking. This reduction in DPPH activity may be associated with heat-related degradation.

Importantly, ORAC and DPPH measure differently in their antioxidant mechanisms—electron transfer versus hydrogen atom transfer, respectively—thus reflecting the activity of distinct phenolic subgroups. 

These results are consistent with previous studies that have analysed the antioxidant activity of individual ingredients. Chickpeas provide phenolic acids and flavonoids with known antioxidant effects, particularly after thermal processing [53]. Similarly, dark chocolate, rich in polyphenols, has also demonstrated a strong antioxidant capacity in both in vitro and clinical studies [54,55].

Although the beneficial properties of these ingredients are well established, to our knowledge, no studies have yet investigated the synergistic effect of a chickpea- and dark chocolate-based snack after in vitro gastrointestinal digestion on total polyphenol content (TPC) and antioxidant activity.

Findings from related studies support the notion that in vitro digestion can enhance the bioaccessibility of phenolic compounds. For instance, recent research on a chickpea flour snack enriched with black carrot pomace (BCP) reported a significant post-digestion increase in the TPC, attributed to the enzymatic release of matrix-bound phenolic compounds during gastrointestinal digestion [56]. Although the antioxidant activity (ABTS and DPPH) decreased after digestion in that study, a strong correlation between the TPC and ABTS was maintained throughout all phases—similar to what we observed in our samples.

These findings support the idea that in vitro digestion enhances the bioaccessibility of polyphenols, even though the measured antioxidant capacity may vary depending on the assay used and the digestion phase. The higher total polyphenol content and antioxidant activity observed in the digested snack samples can be attributed to the presence of dark chocolate. This aligns with findings by Jacimovic et al., who highlighted the health benefits of cocoa-rich dark chocolate, including its high content of bioactive compounds and its antioxidant and anti-inflammatory properties.

In our case, the higher TPC and antioxidant activity observed in the digested snack may largely be attributed to the dark chocolate component. Martini et al. [57] showed that gastrointestinal digestion increases the bioaccessibility of phenolic compounds in dark chocolate, with a total release of 68.7% at the end of digestion and, even with limited systemic bioavailability, can reduce oxidative stress locally in the intestinal tract by neutralising reactive oxygen species.

Although systemic bioavailability may be limited, these compounds can act locally in the gut to neutralise reactive oxygen species and reduce oxidative stress [57,58,59].

While our results confirm the beneficial antioxidant potential of the digested snack matrix, a methodological limitation must be recognised. Although this study did not quantify polyphenols in each individual phase of in vitro digestion, the focus of the analysis was to study the final bioaccessible fraction collected after the intestinal phase, which is generally considered the most physiologically relevant, as it contains compounds potentially available for absorption.

Moreover, it is well known that pH variations during the different stages of digestion can significantly affect the stability and release of polyphenols. Acidic gastric conditions can cause the degradation of some compounds, while neutral to slightly alkaline intestinal conditions can increase their solubility and availability [60].

Given these promising antioxidant results and the known local effects of dietary polyphenols at the intestinal level, we next investigated whether the digested products could exert protective and anti-inflammatory properties using an in vitro intestinal model.

In recent years, in vitro models have gained attention in toxicological research due to ethical and scientific motivations. Among these models, differentiated Caco-2 cells are widely used stand out for their superior morphological and functional differentiation into enterocyte-like cells compared to other colon carcinoma cell lines. However, the literature reports significant discrepancies among different Caco-2 cell lines, which complicates the comparison of the results. This variability is due to differences in culture conditions, serum type, media supplements, passage number, clone origin, and other various parameters, complicating the comparison of the results [29]. Therefore, it is essential to standardise the Caco-2 cell intestinal epithelium model and develop validated testing protocols to improve reproducibility [35]. The literature shows a wide range of opinions on the ideal timing after seeding to achieve a reliably differentiated culture for experiments. Some researchers begin using Caco-2 cells as early as 6–9 days post-seeding [61], while others wait 30 days to yield better results [62].

For all these reasons, with the aim of setting the best conditions to explore the anti-inflammatory effect of all digested products on an intestinal epithelium model, we tested two seeding densities (1 × 10^5^/cm^2^ and 1 × 10^5^/well) and evaluated epithelial development after 15 and 21 days. Based on the results obtained, the gene expression of differentiation markers (ALPI, SLC11A2, and SLC15A1) and tight junction proteins (ZONULIN1, OCCLUDIN, and CLAUDIN1) indicated that 15 days post-seeding was the optimal time point, as expression levels declined by day 21. ALPI and SLC11A2 showed significant upregulation at 15 days, particularly at the higher seeding density, while CLAUDIN1 and ZONULIN1 were significantly elevated at the lower density. These findings are consistent with previous studies reporting peak epithelial differentiation around day 14–15 [29]. To further assess epithelial integrity, TEER and Lucifer Yellow assays were performed. While Lucifer Yellow did not show significant differences in permeability between the seeding densities at 15 or 21 days, TEER measurements were more informative. The density of 1 × 10^5^ cells/cm^2^ showed an initial increase in resistance followed by a decline after day 10, indicating early epithelial degradation. In contrast, the 1 × 10^5^ cells/well condition showed a steady increase in TEER, exceeding 150 Ω-cm^2^ from day 7 and remaining stable for 17 days.

Although both TEER measurements and LY can be used to evaluate tight junction integrity, they assess different aspects. TEER evaluate ionic resistance across the epithelial monolayer, while LY assesses paracellular permeability based on non-electrolyte flux [34]. In our study, LY did not differentiate between conditions, suggesting it may lack sensitivity for detecting subtle changes. 

TEER is often considered more reliable for assessing the integrity of the epithelium [46,47]. This is because TEER directly measures electrical resistance across the cell monolayer, providing a quantitative estimate of the cohesion and integrity of cell junctions. These results confirmed that the 15-day culture using 1 × 10^5^ cells/well was identified as the most suitable condition for establishing a well-formed and functionally differentiated intestinal epithelium.

The intestinal barrier plays an important role in the protection of the whole organism against toxic substances, maintaining gut health, and preventing disease [35]. Moreover, the mucosal barrier acts as the first line of defence for gut contents and the body’s tissues. Damage to this barrier can enable pro-inflammatory molecules to pass through, leading to an overactive immune response, chronic inflammation, and tissue damage. Increased intestinal permeability is associated with several inflammatory conditions.

A diet rich in anti-inflammatory foods is key to protecting the intestinal barrier and reducing inflammation. Chickpeas, particularly, stand out for their nutritional benefits and positive impact on gut health and barrier integrity [20].

This evidence suggests that chickpeas, possibly due to their richness in bioactive compounds, may play a crucial role in maintaining and protecting intestinal epithelial integrity. Their polyphenols have demonstrated significant antioxidant and anti-inflammatory properties, which are essential in mitigating intestinal inflammation and enhancing barrier function. Studies have shown that these compounds can also modulate inflammatory pathways and promote a healthy gut microbiome, thereby strengthening the epithelial barrier by improving the expression and function of tight junction proteins [63].

In our study, digested chickpea, dark chocolate, and snack products demonstrated a dual protective effect on gut health, simultaneously preserving barrier integrity and modulating inflammatory responses. Pre-treatment of Caco-2 monolayers with the digested products significantly prevented the reduction in TEER values typically induced by inflammatory stimuli (LPS and IL-1β), indicating the preservation of epithelial tightness. This protective effect was further supported at the molecular level, as demonstrated by the upregulation of tight junction genes such as ZO-1, Claudin-1, and Occludin, particularly after treatment with digested chickpea products.

As shown by Ulluwishewa et al. [64], polyphenols increase TEER across Caco-2 monolayers in a dose-dependent manner and promote tight junction assembly in Caco-2 cells [65]. Amasheh et al. also found that polyphenols from chickpeas improved TEER of Caco-2 cell monolayers by increasing Claudin levels [66] and protecting the integrity of HT-29/B6 cell monolayers from TNF-α-induced damage [67]. Similarly, Suzukia et al. reported that polyphenols showed protective and promotive effects on intestinal tight junction barrier function by regulating tight junction protein expression [68].

In parallel, the reduction in inflammatory markers supported the beneficial role of these food matrices. In Caco-2 cells, treatment with digested chickpea and chocolate significantly reduced IL-1β, a key pro-inflammatory cytokine involved in barrier disruption.

Additionally, a notable study examined how chocolate helps to preserve the integrity of the epithelial barrier, potentially through its ability to inhibit the apoptosis induced by oxysterols [69]. It may also help reduce inflammation associated with obesity by lowering MCP-1 and IL-1β levels, which are involved in adipocyte development. Chocolate’s primary mechanism seems to be the suppression of inflammatory mediators, like IL-1β, which typically compromise the structural stability of cell membranes [69]. This aligns with our findings, where chocolate not only preserved the intestinal barrier under inflammatory conditions but also significantly reduced IL-1β expression.

Furthermore, in THP-1 immune cells, exposure to the digested products increased the expression of anti-inflammatory markers such as IL-10 and CD206, indicating a shift to a M2-like macrophage phenotype associated with tissue repair and inflammation resolution.

Specifically, in our study, chocolate showed anti-inflammatory properties by increasing the expression of the CD206 and IL-10 genes. Similar effects were observed for the snack formulation at concentrations of 15.1 mg/mL and 30.3 mg/mL.

Based on previous studies, CD206 and IL-10 act as markers for M2 macrophages, known for their role in reducing inflammation and anti-inflammatory functions. Previous studies have established CD206 and IL-10 as reliable markers of M2 macrophages, which exert anti-inflammatory functions. Genine et al. reported that the THP-1/M2 phenotype expressed several key markers, including CD206, CD163, fibronectin, IL-10, CCL18, and CCL22, with a modest increase in CD206 and IL-10 observed after 24 h of incubation. In fact, after a 24 h incubation period, both CD206 and IL-10 showed a slight increase in THP-1/M2 cells [70]. If CD206 gene expression increases following cocoa treatment in THP-1 cells, this could indicate a shift towards M2 macrophage polarisation, suggesting that chocolate, probably due to its polyphenols and bioactive components, promotes an anti-inflammatory response of M2 macrophages [70]. Our results are consistent with those of Sarria et al., who reported that cocoa consumption reduced both modulated IL-1β and IL-10 levels, suggesting a decrease in inflammatory cytokines, also evaluated in a snack digested product for the contribution and the presence of chocolate [71].

This dual downregulation was interpreted as a general reduction in inflammatory signalling, consistent with our observation of decreased levels of IL-1β and modulated IL-10 levels following treatment with the snack containing chocolate. Taken together, these data suggest that chocolate may exert a dual role: supporting anti-inflammatory polarisation through M2 macrophage markers while also contributing to a downregulation in inflammatory cytokines. This dual downregulation was consistent with our observation of decreased IL-1β expression and modulated IL-10 levels following treatment with the snack matrix, supporting the hypothesis that chocolate contributes to both anti-inflammatory macrophage polarisation and a reduction in inflammatory cytokines.

Interestingly, while chocolate exerted an anti-inflammatory effect, digested chickpeas showed a pro-inflammatory response induced by under these experimental conditions. Specific studies have shown that chickpeas, especially following digestion, may activate inflammatory pathways such as NF-κB, leading to the upregulation of cytokines including IL-8, IL-6, and TNF-α [72,73,74].

Additionally, other studies demonstrated that the interaction with gut microbiota might influence immune responses that elevate cytokine levels, including IL-8, in intestinal cells [63,74].

These findings support our results, where chickpea and snack digested products increased IL-8 gene expression. However, this pro-inflammatory signal may vary depending on digestion processes and the bioavailability of specific peptides. Despite this evidence, our findings indicate that digested chickpeas possess a peculiar ability to enhance epithelial integrity by upregulating tight junction protein expression, suggesting a dual role.

In addition, the antioxidant capacity observed in the digested products, particularly in the snack and chocolate matrices, may have contributed to the maintenance of epithelial barrier integrity. Oxidative stress is a well-known trigger of tight junction disruption and epithelial permeability, and the antioxidant compounds present in the chickpea- and dark chocolate-based snack could have mitigated oxidative damage at the intestinal level. Thus, the combination of antioxidant activity and anti-inflammatory modulation likely acted synergistically to preserve gut barrier function under inflammatory conditions.

Although the specific bioactive compounds responsible for the observed effects were not characterised in this study, the results highlight the functional potential of the chickpea- and dark chocolate-based snack.

Taken together, these results support the hypothesis that chickpeas and dark chocolate can contribute to gut health through distinct but complementary mechanisms. Their combination within a single food matrix may promote mechanistic synergy, enhancing the protective effect on the intestinal epithelium more effectively than either component alone.

This hypothesis aligns with the widely accepted concept of the “whole food effect,” which recognises that the health benefits of many plant-based foods arise not from a single compound, but from the synergistic interaction of multiple constituents within the food matrix, such as fibres, polyphenols, and other phytochemicals [75]. This concept recognises the importance of studying foods as complex systems where interactions among nutrients and bioactive molecules may enhance biological activities. In this context, the protective effects on intestinal barrier integrity and modulation of inflammatory responses observed in this work could be attributed to the combined influence of the various bioactive constituents naturally present in chickpeas and dark chocolate.

However, it is important to emphasise that this study was able to comprehensively characterise the nature of the interaction between the two food components.

Although our results suggest that combining dark chocolate and chickpeas at half the individual doses in a single food matrix may result in effects comparable to those observed with full doses of the individual components, this observation alone is not sufficient to definitively define the interaction as additive or synergistic. The complexity of food matrices and the possibility of non-linear biological responses make it difficult to isolate the contribution of each component. Furthermore, the variability between different biomarkers further supports the idea that each component may act on distinct biological pathways.

Together, these findings suggest that the digested products not only act directly on intestinal epithelial cells but also modulate immune activity, thereby promoting intestinal homeostasis by modulating immune cell responses. This combined action highlights the potential of the chickpea-dark chocolate snack as a functional food, capable of supporting gut health and preventing chronic inflammation.

Beyond the scientific findings, it is important to consider the practical applications of the developed chickpea- and dark chocolate-based snack. While this study highlights its potential in promoting intestinal health and modulating inflammatory responses, such evidence may serve as a scientific foundation for further investigations focused on its commercial viability as a functional food.

Consumer acceptance is also a critical factor for the success of innovative food products and plays a pivotal role in the food industry. Understanding sensory preferences, willingness to pay (WTP), and purchasing motivations can help ensure successful market introduction [76]. Although there is no direct consumer acceptance analysis for this product, studies have shown that clear communication of health benefits and product familiarity enhance the acceptance of functional and plant-based foods [77].

Recent consumer research revealed a growing openness to trying novel plant-based products, especially when associated with attributes such as health, sensory pleasure, and sustainability [78]. The gastronomic implications of these findings are significant: the perceptions gathered from diverse consumer profiles may help guide and accelerate the development and popularity of new plant-based snack options.

Moreover, a report on European consumer behaviour indicated a particular interest in legumes—such as chickpeas—with 43% of participants intending to replace animal products, 52% to increase consumption, and 53% to purchase them more regularly [76]. This confirms the increasing potential of chickpea-based products in the European market.

In this scenario, ready-to-eat legume-based snacks are gaining popularity due to their nutritional benefits, palatability, and convenience.

These products fit well with current dietary trends, where traditional meals are being increasingly replaced by portable, nutrient-rich snacks [79]. Furthermore, nutritional guidelines are increasingly recommending the redefinition of “snack foods” to prevent the overconsumption of highly processed, low-nutrient options [80].

Therefore, the functional and nutritional benefits demonstrated in this work align with broader market demands for healthier snack alternatives. The development of a chickpea- and dark chocolate-based snack not only offers potential health benefits but also meets evolving consumer expectations for sustainable, nutritious, and enjoyable food options. These insights support its dual role as a scientifically validated and commercially promising functional food.

## 5. Conclusions

This study provides evidence that a snack combining puffed chickpeas and dark chocolate exerts beneficial effects on intestinal barrier function and immune modulation in vitro. Chickpeas mainly contributed to strengthening epithelial integrity, while dark chocolate appeared to support anti-inflammatory and antioxidant mechanisms. The integration of these two ingredients in a single food matrix may promote mechanistic synergy, with each component acting on different but complementary biological pathways. Although further research on more complex models is needed, these results support the potential of this snack as a functional food capable of acting on multiple aspects of gut and immune health through the combined action of its bioactive components.

Limitations of this study include the reliance on simplified in vitro models such as Caco-2 cells, which do not fully reproduce the complexity of the human gut, and the focus on a limited set of inflammatory markers. Future studies supporting the findings at the level of proteins (e.g., Western blot and immunofluorescence) with cytokine secretion data (e.g., ELISA) or dose–response experiments and comparisons with known beneficial compounds will enrich this preliminary analysis. Moreover, the lack of quantitative analysis of the total polyphenol content at each stage of in vitro digestion limits a more detailed understanding of polyphenol release kinetics and their pH-dependent stability during digestion.

Ongoing research will incorporate more advanced gut models that include interactions with the microbiota and broader immune pathways to better reflect in vivo conditions. Despite the inherent limitations of simplified in vitro systems, these findings suggest promising nutrigenomic potential for functional foods in promoting gut and immune health. Future studies using more complex models will help to confirm these findings and investigate additional underlying mechanisms.

## Data Availability

Data is contained within the article and supplementary material.

## Supplementary Materials

The following supporting information can be downloaded at https://www.mdpi.com/article/10.3390/antiox14070823/s1, Table S1: Primers for tight junctions’ genes, for genes of differentiation in Caco-2 cells, for inflammatory genes and anti-inflammatory genes for THP-1, Table S2: Pearson’s correlation coefficient between TPC and antioxidant activities of digested products, Figure S1: Expression levels of genes involved in Caco-2 differentiation. ALPI (A), SLC15A1 (B), SLC11A2 (C) expression levels, measured with the qPCR, on Caco-2 intestinal epithelium model for each density evaluated: 1 × 10^5^/cm^2^ after 15 days (15D) and 21 days (21D) post-seeding, 1 × 10^5^/well after 15D and 21D post-seeding, Figure S2: Expression levels of genes involved in the formation of tight junctions. CLAUDIN1 (A), ZONULIN1 (B), OCCLUDIN (C) expression levels, measured with the qPCR, on Caco-2 intestinal epithelium model for each seeding density evaluated: 1 × 10^5^/cm^2^ 15D and 21D, after 15 days and 21 days post-seeding, 1 × 10^5^/well 15D and 21D, after 15 days and 21 days post-seeding, Figure S3: Paracellular permeability of LY through Caco-2 monolayers cells after 15 days (A) and 21 (B) days post-seeding of the two different seeding densities (1 × 10^5^/cm^2^ and 1 × 10^5^/well), Figure S4: Barrier integrity performed with TEER in cells cultured in trans-wells using two different densities of seeding (1 × 10^5^/cm^2^, A, and 1 × 10^5^/well, B) during 17 days of seeding to evaluate a well-formed epithelium, Figure S5: Evaluation of barrier integrity by transepithelial electrical resistance (TEER) in Caco-2 cells cultured in trans-well inserts.

## Abbreviations

The following abbreviations are used in this manuscript:

MUFAs	Monounsaturated fatty acids
PUFAs	Polyunsaturated fatty acids
GI	Glycaemic index
Caco-2 cells	Colon adenocarcinoma cells
SGF	Simulated stomach fluid
SIF	Simulated intestinal fluid
TPC	Total polyphenol content
GAE	Gallic acid equivalents
DPPH	2,2-diphenyl-1-picrylhydrazyl
ABTS	2,2′-azino-bis (3-ethylbenzothiazoline-6- sulphonic acid)
K_2_S_2_O_8_	Potassium persulphate
ORAC	Oxygen radical absorbance capacity
AUC	Area under the curve
DMEM	Dulbecco’s modified Eagle’s medium
FBS	Foetal bovine serum
NEAAs	Non-essential amino acids
P/S	Penicillin/streptomycin
MTT	3-(4,5-Di-2-yl)-2,5-ditetrazolium bromide
PBS	Phosphate-buffered saline
AP	Apical
BL	Basal
IL-1β	Interleukin 1β
LPS	Lipopolysaccharide
TEER	Transepithelial electrical resistance
LY	Lucifer Yellow
THP-1	Human acute monocytic leukaemia cell line
PMA	Phorbol-12-myristate-13- acetate (PMA), 1α
vD3	25-dihydroxyvitamin D3
M-CSF	Macrophage colony-stimulating factor
RPMI-1640	Roswell Park Memorial Institute 1640 medium
cDNA	Complementary DNA
qPCR	Quantitative real-time polymerase chain reaction
PPIA	Peptidylprolyl Isomerase A
ZO1	Zonulin 1
OCLN	Occludin
CLDN1	Claudin1
ALPI	Alkaline phosphatase intestinal
CYP3A4	Cytochrome P450 Family 3 Subfamily A Member 4
SLC15A1	Solute Carrier Family 15 Member 1
SLC11A2	Solute Carrier Family 11 Member 2
SI	Sucrase-isomaltase
IL-6	Interleukin-6
NF-κB	Nuclear factor kappa-light-chain-enhancer of activated B cells
IL-8	Interleukin 8
MCP1 or CLL2	Monocyte Chemoattractant Protein 1
CCL20	Chemokine (C-C motif) Ligand 20
TNF-α	Tumour necrosis factor alpha
CD206	Mannose receptor C-type 1
IL-10	Interleukin 10
TGFβ	Transforming growth factor beta
PPAR-γ	Peroxisome proliferator-activated receptor gamma
CD163	Cluster of Differentiation 163
BCP	Black carrot pomace
WTP	Willingness to pay
