# Supplementary material for: Explorative Analysis of Antioxidant, Anti-Inflammatory, and Intestinal Barrier Protective Effects of In Vitro Digested Chickpea- and Dark Chocolate-Based Snack: Insights from Caco-2 and THP-1 Cell Models
Source: Antioxidants (Basel). 2025 Jul 4;14(7):823. doi: 10.3390/antiox14070823 (PMC12291799; doi:10.3390/antiox14070823)
Supplement: Supplementary file 1 [file antioxidants-14-00823-s001.zip › antioxidants-3702649-supplementary.pdf]

Supplementary materials for *Evaluation of Antioxidant, Anti-Inflammatory, and Intestinal Barrier Protective Effects of an In Vitro Digested Chickpea and Dark Chocolate-Based Snack: Insights from Caco-2 and THP-1 Cell Models*

Gaia de Simone [1, 2], Laura Bonfili[3], Anna Maria Eleuteri[3], Laura Bordoni*[2] and Rosita Gabbianelli[2*].

[1] School of Advanced Studies, University of Camerino, 62032 Camerino, Italy; gaia.desimone@unicam.it (G.d.S.)

[2] Unit of Molecular Biology and Nutrigenomics, School of Pharmacy and Health Products, University of Camerino, 62032 Camerino, Italy; gaia.desimone@unicam.it (G.d.S.); laura.bordoni@unicam.it (L.B.); rosita.gabbianelli@unicam.it (R.B.)

[3] School of Biosciences and Veterinary Medicine, University of Camerino, Via Gentile III da Varano, 62032 Camerino MC, Italy; annamaria.eleuteri@unicam.it (A.M.E); laura.bonfili@unicam.it (L.BO.)

* Correspondence: rosita.gabbianelli@unicam.it (R.G.); Tel.: +39-0737-403208; laura.bordoni@unicam.it (L.B.); Tel.: +39-0737-403211

	Fw (5'-3')	Rv (5'-3')
**CD206 (C-type mannose receptor 1)**	TAT GGA ATA AAG ACC CGC TGAC	TGC TCA TGT ATC TCT GTG ATGCT
**IL10 (Interleukin-10)**	AGG CAT TCT TCA CCT GCT CC AAG	ACC CAG ACA TCA AGG CG
**TGF-β (Transforming Growth Factor Beta)**	CCC AGC ATC TGC AAA GCT GTC	AAT GTA CAG CTG CCG CA
**PPAR-γ (Peroxisome Proliferator-Activated Receptor Gamma)**	TGT GGG GAT AAA GCA TCA GGC	CCG GCA GTT AAG ATC ACA CCTAT
**CD163 (Cluster of Differentiation 163)**	ACT GCA AGA ACT GGC AAT GG	CCA TGC TTC ACT TCA ACA GG
**βactin**	TGAGAGGGAAATCGTGCGTG	TGCTTGCTGATCCACATCTGC
**IL-6 (Interleukin-6)**	TGC AAT AAC CAC CCC TGA CC	GTG CCC ATG CTA CAT TTG CC
**IL-1β (Interleukin 1β)**	AGA TGA TAA GCC CAC TCT ACAG	ACA TTC AGC ACA GGA CTC TC
**NFKB Nuclear Factor Kappa B**	ACA GCT GGA TGT GTG ACT GG TCC	TCC TCC GAA GCT GGA CAA AC
**IL-8 (Interleukin-8)**	GGA CAA GAG CCA GGA AGA AA	CCT ACA ACA GAC CCA CAC AATA
**MCP1 or CCL2 (Monocyte Chemoattractant Protein 1)**	GGCTGAGACTAACCCAGAAAAG	GGGTAGAAACTGTGGTTACCGAG
**ALPI (Alkaline Phosphatase, Intestinal)**	CATACCTGGCTCTGTCCAAGA	GTCTGGAAGTTGGCCTTGAC
**CYP3A4 (Cytochrome P450 Family 3 Subfamily A Member 4)**	GATGGCTCTCATCCCAGACTT	AGTCCATGTGAATGGGTTCC
**SLC15A1 (Solute Carrier Family 15 Member 1)**	TCTCTGTCACGGGATTGGA	CTGCCTGAAGCACCGACT
**SLC11A2 (Solute Carrier Family 11 Member 2)**	CACCGTCAGTATCCCAAGGT	CCGATGATAGCCAACTCCAC
**SI (Sucrase-Isomaltase)**	AATCCTTTTGGCATCCAGATT	GCAGCCAAGAATCCCAAAT
**ZO1 (Zonulin1)**	TTCACGCAGTTACGAGCAAG	TTGGTGTTTGAAGGCAGAGC
**OCLN (Occludin)**	GGGCATTGCTCATCCTGAAG	GCCTGTAAGGAGGTGGACTT
**CLDN1 (Claudin1)**	TGGTCAGGCTCTCTTCACTG	TTGGATAGGGCCTTGGTGTT
**PPIA (Peptidylprolyl Isomerase A)**	ATGCTGGACCCAACACAAAT	TCTTTCACTTTGCCAAACACC

**CCL20 (Chemokine (C-C motif) Ligand 20)**	GCG GCG AAT CAG AAG CAA GC	GCA TTG ATG TCA CAG CCT TCA TTG GCC AG
**TNFalfa (Tumor Necrosis Factor Alpha)**	AAG CCT GTA GCC CAC GTG TA	GGC ACC ACT AGT TGG TGG TCT TTG

Table S1. Primers for tight junctions' genes, for genes of differentiation in Caco-2 cells, for inflammatory genes and anti-inflammatory genes for THP-1

Sample	TPC with-	Spearman's Correlation	Sig. 2-Tailed
**Chickpea, dark chocolate and snack digested products**	DPPH	0.262	$p>0.05$
	ABTS	0.727	$p<0.001$
	ORAC	0.572	$p<0.001$

Table S2. Pearson's correlation coefficient between TPC and antioxidant activities of digested products

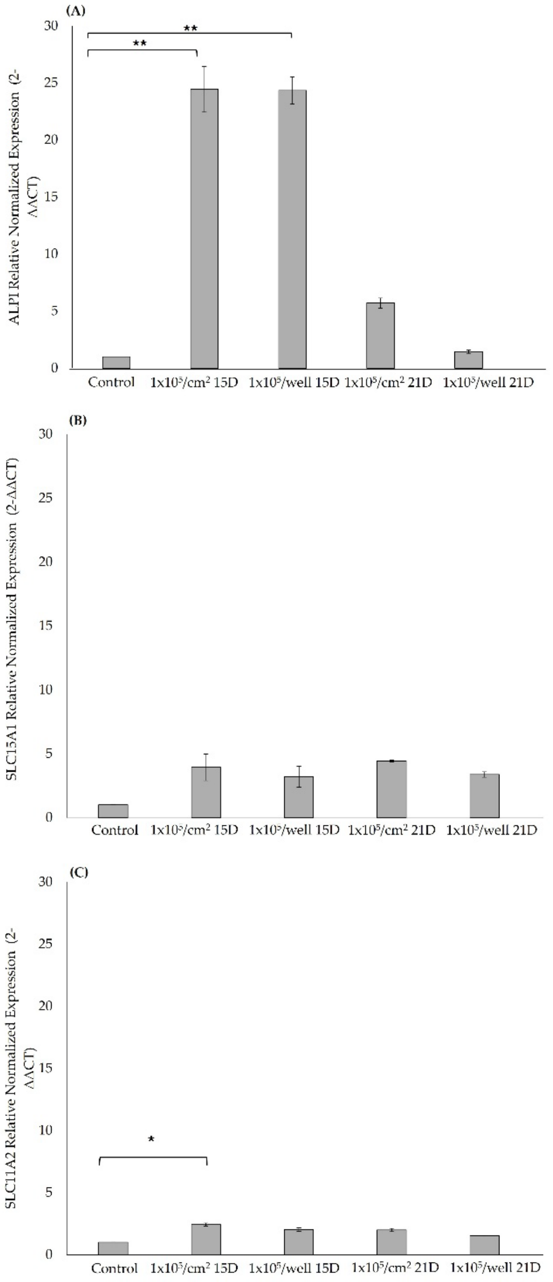

Fig. S1. Expression levels of genes involved in Caco-2 differentiation. ALPI (A), SLC15A1 (B), SLC11A2 (C) expression levels, measured with the qPCR, on Caco-2 intestinal epithelium model for each density evaluated: $1x10^5/cm^2$ after 15 days (15D) and 21 days (21D) post seeding, $1x10^5$/well after 15D and 21D post-seeding. *$p < 0.05$; **$p < 0.01$; vs Control. The existence of significant differences among the different parameters in the samples was evaluated using the non-parametric Kruskal-Wallis test.

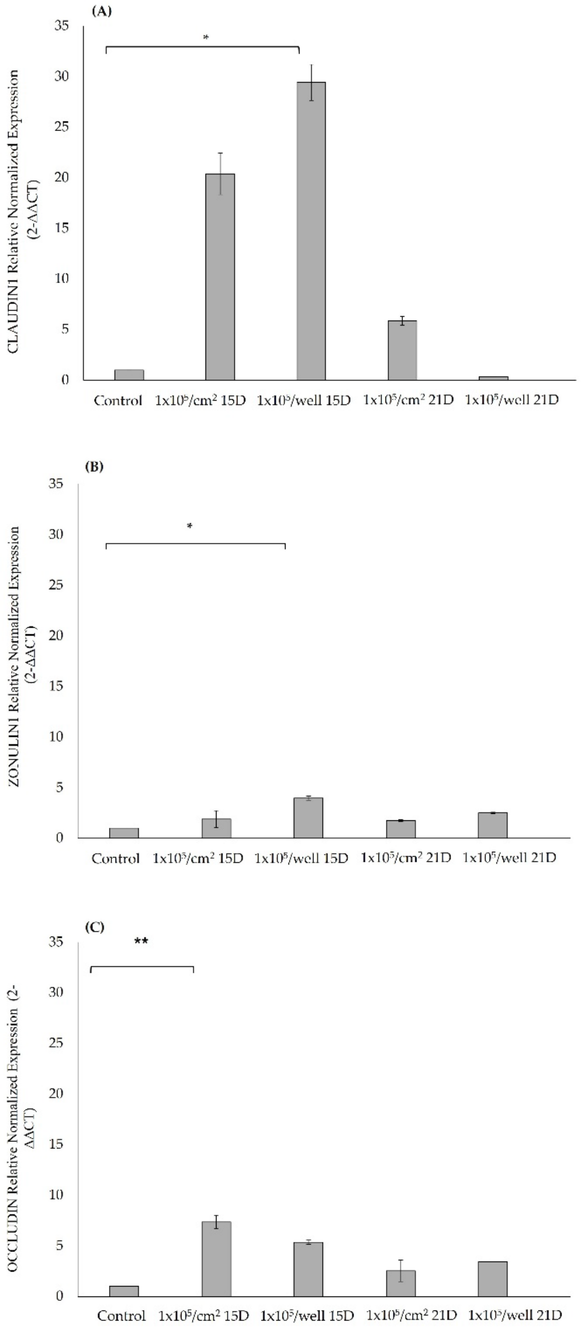

Fig. S2. Expression levels of genes involved in the formation of tight junctions. CLAUDIN1 (A), ZONULIN1 (B), OCCLUDIN (C) expression levels, measured with the qPCR, on Caco-2 intestinal epithelium model for each seeding density evaluated: $1x10^5$/ cm^2 15D and 21D, after 15 days and 21 days post seeding, $1x10^5$/well 15D and 21D, after 15 days and 21 days post-seeding. *$p < 0.05$; **$p < 0.01$;* vs Control. The existence of significant differences among the different parameters in the samples was evaluated using the non-parametric Kruskal-Wallis test.

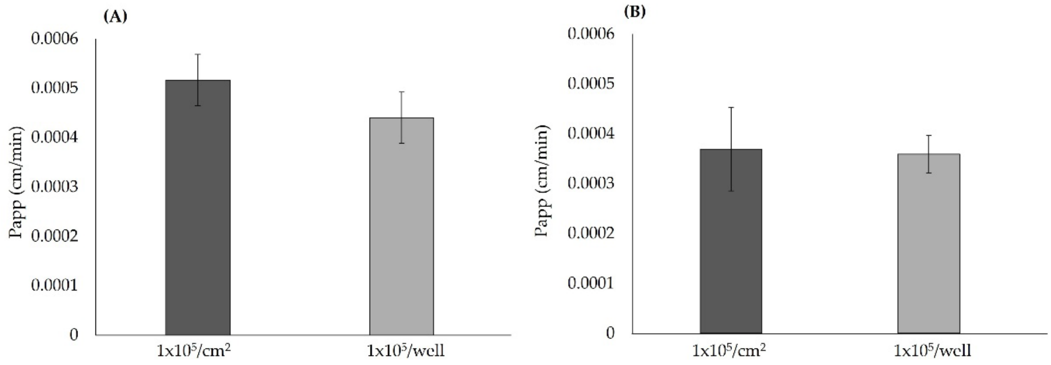

Fig. S3. Paracellular permeability of LY through Caco-2 monolayers cells after 15 days(A) and 21 (B) days post-seeding of the two different seeding densities ($1x10^5/cm^2$ and $1x10^5$/well). LY transport expressed as the instantaneous permeability (Papp). No variations of permeability of the intestinal epithelium were observed in any densities evaluated after 15- and 21-days post seeding. Data are expressed as mean ± SD from three independent experiments. ANOVA test with Bonferroni's correction were used to test differences

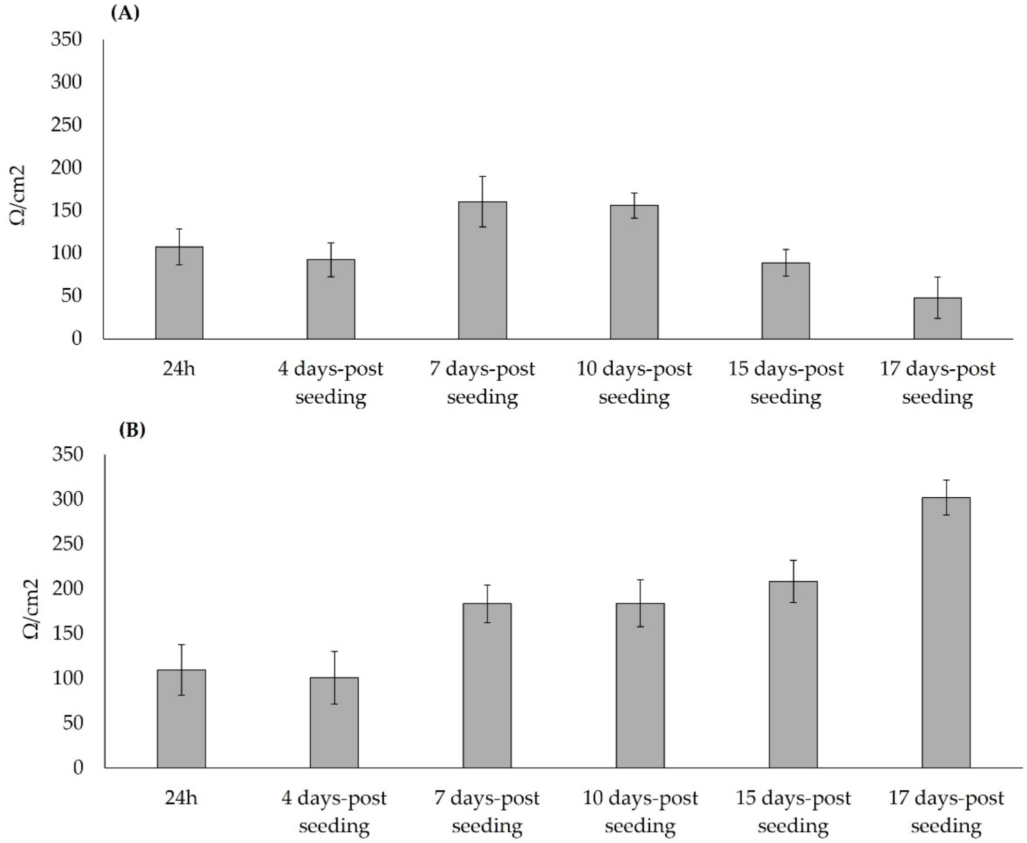

Fig. S4. Barrier integrity performed with TEER in cells cultured in trans-wells using two different densities of seeding ($1x10^5/cm^2$, A, and $1x10^5$/well, B) during 17 days of seeding to evaluate a well-formed epithelium. ANOVA test with Bonferroni's correction were used to test differences.

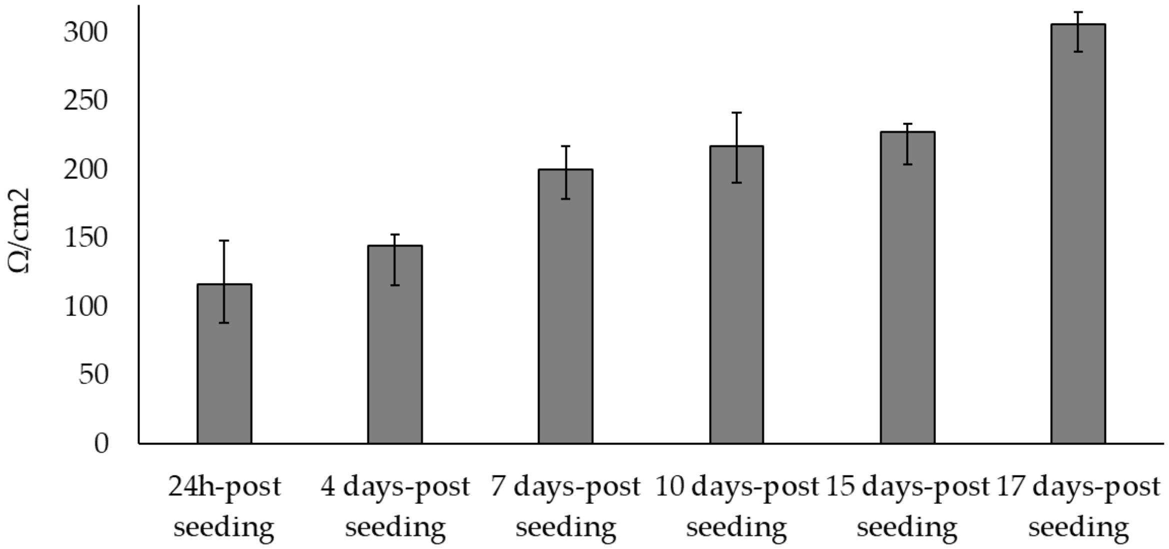

Fig. S5. Evaluation of barrier integrity by transepithelial electrical resistance (TEER) in Caco-2 cells cultured in transwell inserts. Cells were seeded at a density of 1×10^5 cells/well and monitored for 17 days to assess the formation of a functional intestinal epithelium. ANOVA test with Bonferroni's correction were used to test differences
